# Robust Support Vector Data Description with Truncated Loss Function for Outliers Depression

**DOI:** 10.3390/e26080628

**Published:** 2024-07-25

**Authors:** Huakun Chen, Yongxi Lyu, Jingping Shi, Weiguo Zhang

**Affiliations:** Department of Automatic Control, Northwestern Polytechnical University, Xi’an 710072, China; chenhuakun@mail.nwpu.edu.cn (H.C.); shijingping@nwpu.edu.cn (J.S.); zhangwg@nwpu.edu.cn (W.Z.)

**Keywords:** SVDD, truncated loss function, fast ADMM, proximal operators, anomaly detection, truncated binary cross entropy loss function, truncated linear exponential loss function

## Abstract

Support vector data description (SVDD) is widely regarded as an effective technique for addressing anomaly detection problems. However, its performance can significantly deteriorate when the training data are affected by outliers or mislabeled observations. This study introduces a universal truncated loss function framework into the SVDD model to enhance its robustness and employs the fast alternating direction method of multipliers (ADMM) algorithm to solve various truncated loss functions. Moreover, the convergence of the fast ADMM algorithm is analyzed theoretically. Within this framework, we developed the truncated generalized ramp, truncated binary cross entropy, and truncated linear exponential loss functions for SVDD. We conducted extensive experiments on synthetic and real-world datasets to validate the effectiveness of these three SVDD models in handling data with different noise levels, demonstrating their superior robustness and generalization capabilities compared to other SVDD models.

## 1. Introduction

Anomaly detection refers to the identification of data points in a dataset that deviate from normal behavior. These deviations are known as anomalies or outliers in various application domains. This mode of detection is extensively used in real-world settings, including credit card detection, insurance detection, cybersecurity intrusion detection, error detection in security systems, and military activity monitoring [1,2]. However, the acquisition of anomaly data in practical applications, such as medical diagnostics, machine malfunction detection, and circuit quality inspection, is expensive [3,4]. Consequently, there is significant interest in one-class classification (OCC) problems, where training samples only include normal data (also known as target data) or normal data with a small number of anomalies (also referred to as non-target data) [5,6,7]. In this context, it is important to define the following terms:

- Normal data: Normal data refers to data points that conform to the characteristics and behavior patterns of the majority of data points within a dataset. They represent the normal operating state of a system or process.
- Anomalies: Anomalies are data points that significantly deviate from normal patterns and usually reflect actual problems or critical events in the system.
- Outliers: Outliers are data points that are significantly different from other data points in the dataset, which may be due to natural fluctuations, special circumstances, or noise.
- Noise: Noise refers to irregular, random errors or fluctuations, usually caused by measurement errors or data entry mistakes, and does not reflect the actual state of the system.

Support vector data description (SVDD) is a method extensively used for one-class classifications (OCCs) [8]. The core idea of SVDD is to construct a hyper-sphere of minimal volume that encompasses all (or most) of the target class samples. Data points inside the hyper-sphere are considered normal, while those outside are considered anomalies. SVDD can be easily integrated with popular kernel methods or some deep neural network models [7], making it highly scalable and flexible. Due to these attractive features, SVDD has garnered significant attention and has been extensively developed. SVDD is regarded as an effective and excellent technique for anomaly detection problems; however, it remains sensitive to outliers and noise present in training datasets. In real-world scenarios, various issues, such as instrument failure, formatting errors, and unrepresentative sampling, result in datasets with anomalies, which degrade the performance of the SVDD [9,10].

The existing methods for mitigating the impact of noise are typically categorized as follows:

In order to reduce the impact of outliers on the OCC method, researchers have attempted to remove outliers through data preprocessing methods. Stanley Fong used methods such as cluster analysis to remove anomalies from the training set to achieve a robust classifier [11]. Breunig et al. tried to assign an outlier score to each sample in the dataset by estimating its local density, known as LOF [12]. Zheng et al. used LOF to filter raw samples and remove outliers [13]. Khan et al. [14] and Andreou and Karathanassi [15] calculated the interquartile range (IQR) of training samples, which provides a method for indicating a boundary beyond which samples are marked as outliers and removed. Clustering (k-means, DBSCAN) or LOF methods can significantly reduce noise points in data preprocessing, thereby improving the quality and effectiveness of subsequent model training. For datasets with obvious noise and significant distribution characteristics, preprocessing methods can be very effective in enhancing model performance. However, preprocessing methods have several issues: they require additional computational resources and time, especially with large datasets, potentially making the preprocessing step time-consuming. Moreover, there is a risk of overfitting and inadvertently deleting useful normal data points, impacting the model’s ability to accurately detect anomalies. Additionally, these preprocessing methods are sensitive to parameter settings, necessitating careful selection to achieve satisfactory results.

Zhao et al. proposed the dynamic radius SVDD method that accounts for hyper-sphere radius information and the existing data distribution [16,17]. This approach achieves a more flexible decision boundary by assigning different radii to different samples. Moreover, the framework for the dynamic radius SVDD method is based on the traditional SVDD framework. However, if the traditional SVDD has not undergone adequate training, the good performance of these dynamic approaches will be difficult to guarantee.

Density-weighted SVDD, position-weighted SVDD, Stahel–Donoho outlier-weighted SVDD, global plus local joint-weighted SVDD, and confidence-weighted SVDD are examples of weighted methods [18,19,20,21,22,23,24]. These methods assign smaller weights to sparse data, which are commonly outliers, thus excluding them from the sphere. These methods balance the target class data and outliers in the training phase, thus enhancing the classification performance, especially when the data are contaminated by outliers. However, when the number of outliers in the dataset increases and they form sparse clusters, the number of outliers might surpass that of normal samples. In such cases, weighted methods assign higher weights to the outliers and lower weights to the normal samples, leading to decreased algorithm performance.

The convex property of the hinge loss function of the SVDD algorithm makes it sensitive to outliers. To address this issue, Xing et al. proposed a new robust least squares one-class support vector machine (OCSVM) that employs a bounded, non-convex entropic loss function, instead of the unbounded convex quadratic loss function used in traditional least squares OCSVM [25]. The non-convex nature of the ramp loss function makes this model more robust than the traditional OCSVM [26]. Tian et al. introduced the ramp loss function to the traditional OCSVM to create the Ramp-OCSVM model [27], and the non-convex nature of this model makes it more robust than the traditional OCSVM. Xing et al. enhanced the robustness of the OCSVM by introducing a re-scaled hinge loss function [28]. Additionally, Zhong et al. proposed a new robust SVDD method, called pinball loss SVDD [29], to perform OCC tasks when the data are contaminated by outliers. The pinball loss function ensures minimal dispersion at the center of the sphere, thus creating a tighter decision boundary. Recently, Zheng introduced a mixed exponential loss function to the design of the SVDD model, enhancing its robustness and making its implementation easier [30].

Extensive research has shown that, as a result of unbounded convex loss functions being sensitive to anomalies, loss functions with boundedness or bounded influence functions are more robust to the influence of outliers. To address this issue, researchers introduced an upper limit to unbounded loss functions, effectively preventing them from increasing beyond a certain point. The truncated loss function thus makes the SVDD model more robust. The advantages of truncated loss functions include:

- Robustness to noise: Truncated loss functions can enhance the model’s robustness and stability by limiting the impact of outliers without removing data points.
- Reduction of error propagation: In anomaly detection tasks, outliers may significantly contribute to the loss function, leading to error propagation and model instability. Truncated loss functions can effectively reduce error propagation caused by outliers, thereby improving overall model performance.
- Generalization ability: Using truncated loss functions can prevent the model from overfitting to outliers, enhancing the model’s generalization ability. Truncated loss functions are well-suited for various types of datasets and noise conditions, particularly when noise is not obvious or easily detectable.

However, robust SVDD algorithms still face considerable challenges in the research. They are designed to address specific types of losses and lack an appropriate framework for constructing robust loss functions. Thus, researchers are required to learn how to use different algorithms and modify loss functions before use. Since truncated loss functions are often non-differentiable, methods such as the difference of convex algorithm (DCA) [31] and concave–convex procedures (CCCPs) [32,33] are commonly employed to provide solutions. For some truncated loss functions, the DCA cannot ensure straightforward decompositions or the direct use of comprehensive convex toolboxes, potentially increasing development and maintenance costs [34]. At present, no unified framework exists in the literature for the design of robust loss functions or a unified optimization algorithm. Therefore, even though this is challenging, providing a new bounded strategy for the SVDD model is crucial, with the potential for developing more efficient and universally applicable solutions.

In response to the several issues previously outlined, this study proposes a universal framework for the truncated loss functions of the SVDD model. To address and solve the non-differentiable, non-convex optimization problem introduced by the truncated loss function, we employ the fast ADMM algorithm. Our contributions to this field of study are as follows:

- We define a universal truncated loss framework that smoothly and adaptively binds loss functions, while preserving their symmetry and sparsity.
- To solve different truncated loss functions, we propose the use of a unified proximal operator algorithm.
- We introduce a fast ADMM algorithm to handle any truncated loss function within a unified scheme.
- We implement the proposed robust SVDD model for various datasets with different noise intensities. The experimental results for real datasets show that the proposed model exhibits superior resistance to outliers and noise compared to more traditional methods.

The remainder of this paper is organized as follows:

Section 2: We review related support vector data description (SVDD) models, providing a foundational understanding of the existing methodologies and their limitations.

Section 3: We propose a general framework for truncated loss functions. Within this framework, we examine the representative loss functions’ proximal operators and present a universal algorithm for solving these proximal operators.

Section 4: This section introduces the SVDD model that utilizes the truncated loss function, detailing its structure and theoretical framework.

Section 5: A new algorithm for solving the SVDD model with truncated loss functions is presented. This section also includes an analysis of the algorithm’s convergence properties, ensuring that the method is both robust and reliable.

Section 6: Numerical experiments and parameter analysis are conducted to validate the effectiveness of the proposed model. This section provides empirical evidence of the model’s performance across various datasets and noise scenarios.

Section 7: The conclusion summarizes the findings and contributions of the study, and discusses potential future research directions.

## 2. Related Works

SVDD has been widely applied in anomaly detection, and numerous learning algorithms based on SVDD have been proposed. In this section, we provide a brief overview of these algorithms.

### 2.1. SVDD

The goal of SVDD is to discover a hyper-sphere that encompasses target samples, while excluding non-target samples located outside it [8]. The objective function of SVDD is represented by the following equation:

(1) $$\begin{array}{l} \underset{R,\mu ,\xi_{i}}{\min}\;R^{2}+C\sum_{i}\xi_{i}\\ s.t\;{\left\|\varphi \left(x_{i}\right)-\mu \right\|}^{2}\leq R^{2}+\xi_{i},\;\xi_{i}>0,\;\forall i \end{array}$$

where μ and R represent the radius and center of the hyper-sphere, C is a regularization parameter, and $\xi_{i}$ is a slack variable. By using Lagrange multipliers and incorporating the constraints into the objective function, the dual problem of Equation (1) can be expressed as:

(2) $$\begin{array}{l} \max\;\sum_{i}a_{i}\left(x_{i}\cdot x_{i}\right)-\sum_{i,j}a_{i}a_{j}\left(x_{i}\cdot x_{j}\right)\\ s.t\;\sum_{i}a_{i}=1,\;0\leq a_{i}\leq C,\;\forall i \end{array}$$

where $\alpha_{i}$ is the Lagrange multiplier, the optimization problem in Equation (2) is a standard quadratic programming problem, and $\alpha_{i}$ can be obtained using quadratic programming algorithms. μ and R can be calculated using the following equation:

(3) $$\mu =\sum_{i=1}^{n}\alpha_{i}\varphi \left(x_{i}\right)$$

(4) $$R^{2}=\left(x_{s}\cdot x_{s}\right)-2\sum_{i}\alpha_{i}\left(x_{s}\cdot x_{i}\right)+\sum_{i,j}\alpha_{i}\alpha_{j}\left(x_{i}\cdot x_{j}\right)$$

where $x_{s}$ represents support vectors. The decision function for the test sample z is presented as follows:

(5) $$f\left(z\right)=sign\left(R^{2}-{\left\|z-\mu \right\|}^{2}\right)=sign\left\{R^{2}-\left(z\cdot z\right)+2\sum_{i}a_{i}\left(z\cdot x_{i}\right)-\sum_{i,j}a_{i}a_{j}\left(x_{i}\cdot x_{j}\right)\right\}$$

If f(z)≤0, then sample z belongs to the target class; otherwise, z is considered a non-target class sample.

### 2.2. Robust SVDD Variants

Due to its sensitivity to outliers, the classification performance of SVDD significantly deteriorates when the data are contaminated. Thus, to enhance the robustness of SVDD, various improved SVDD methods have been proposed over the past decades.

#### 2.2.1. Weighted SVDDs

One common approach is the use of weighted SVDD methods, where different slack variables are assigned different weights [18,19,20,21,22,23,24]. Although the specific methods for weight distribution vary, they can generally be represented in a unified form:

(6) $$\begin{array}{l} \underset{R,\mu ,\xi_{i}}{\min}\;R^{2}+C\sum_{i}w_{i}\xi_{i}\\ s.t\;{\left\|\varphi \left(x_{i}\right)-\mu \right\|}^{2}\leq R^{2}+\xi_{i},\;\xi_{i}>0,\;\forall i \end{array}$$

where ${\left\{w_{i}\right\}}_{i=1}^{n}$ represents pre-calculated weights. The density weighting method permits these weights to be calculated as follows [20]:

(7) $$w_{i}=1-\frac{d\left(x_{i},x_{i}^{k}\right)}{\max\left\{d\left(x_{1},x_{1}^{k}\right),\cdots ,d\left(x_{n},x_{n}^{k}\right)\right\}}$$

where $x_{i}^{k}$ denotes the k-nearest neighbor value of $x_{i}$, and $d\left(x_{i},x_{i}^{k}\right)$ denotes the Euclidean distance between $x_{i}$ and $x_{i}^{k}$. Due to outliers typically being located in relatively low-density areas, the distance to their neighboring samples is greater when compared to normal samples, which results in their smaller weights. The R-SVDD algorithm constructs weights by introducing a local density to each data point based on truncated distances [19].

(8) $$\rho_{i}=\sum_{j}\exp\left(-{\left(\frac{d_{ij}}{d_{c}}\right)}^{2}\right)$$

where $d_{ij}$ denotes the distance between $x_{i}$ and $x_{j}$, and $d_{c}$ is the truncation distance. The calculation of the weight function is as follows:

(9) $$w\left(x_{i}\right)=\frac{\rho \left(x_{i}\right)}{\max\left\{\rho \left(x_{1}\right),\cdots ,\rho \left(x_{n}\right)\right\}}$$

Other methods for calculating can refer to [21,22,23,24]. The dual problem of Equation (6) is represented by the following equation:

(10) $$\begin{array}{l} \max\;\sum_{i}\alpha_{i}\left(x_{i}\cdot x_{i}\right)-\sum_{i,j}\alpha_{i}\alpha_{j}\left(x_{i}\cdot x_{j}\right)\\ s.t\;\sum_{i}\alpha_{i}=1,\;0\leq \alpha_{i}\leq w_{i}C,\;\forall i \end{array}$$

From this equation, it can be observed that each Lagrange multiplier has an upper limit, denoted as $\alpha_{i}\leq w_{i}C$.

#### 2.2.2. Pinball Loss SVDD

The pinball loss SVDD (Pin-SVDD) modifies the hinge loss SVDD by replacing its loss function with the pinball loss function to create an optimized problem formulation [29]. This modification enables a more robust handling of outliers by adjusting the sensitivity toward deviations, depending on their direction relative to the decision boundary.

(11) $$\begin{array}{l} \underset{R,\mu ,\xi_{i}}{\min}\;R^{2}+C\sum_{i}\xi_{i}\\ s.t\;{\left\|\varphi \left(x_{i}\right)-\mu \right\|}^{2}\leq \left(1-\tau \right)R^{2}+\xi_{i},\\ {\left\|\varphi \left(x_{i}\right)-\mu \right\|}^{2}\leq \frac{1}{\tau }\xi_{i},\forall i \end{array}$$

where 0<τ≤1 is a constant. With the use of the Lagrange multipliers method, the dual optimization problem in Equation (11) can be expressed as follows:

(12) $$\begin{array}{l} \max\;\sum_{i}\alpha_{i}\left(g_{1}\left(x_{i}\cdot x_{i}\right)+g_{2}\sum_{j=1}^{n}\left(x_{i}\cdot x_{j}\right)\right)+h\sum_{i,j}\alpha_{i}\alpha_{j}\left(x_{i}\cdot x_{j}\right)\\ s.t\;\sum_{i}\alpha_{i}=\frac{1}{1-\tau },\;0\leq \alpha_{i}\leq C,\;\forall i \end{array}$$

where $h=-\frac{{\left(1-\tau \right)}^{2}}{\tau nC+1}$, $g_{1}=1-\tau$, and $g_{2}=-\frac{2\left(1-\tau \right)\tau c}{\tau nc+1}$. Zhong demonstrated that the use of a pinball to minimize scattering at the center of the sphere enhances the robustness of the developed model.

#### 2.2.3. SVDD with Mixed Exponential Loss Function

Zheng used a mixed exponential loss function to design a classification model, and the optimization problem of the method is presented by the following equation [30]:

(13) $$\begin{array}{l} \underset{R,\mu ,\xi_{i}}{\min}\;R^{2}+C\sum_{i}\rho \left(\xi_{i}\right)\\ s.t\;{\left\|\varphi \left(x_{i}\right)-\mu \right\|}^{2}\leq R^{2}+\xi_{i},\;\xi_{i}>0,\;\forall i \end{array}$$

where $\rho \left(\xi_{i}\right)$ is a mixed exponential function of $\xi_{i}$, expressed as follows:

(14) $$\rho \left(\xi_{i}\right)=\lambda \exp\left(-\tau_{1}\xi_{i}\right)+\left(1-\lambda \right)\exp\left(-\tau_{2}\xi_{i}\right)$$

where $\tau_{1}>0$ and $\tau_{2}>0$ are two scale parameters, and 1≥λ≥0 is a mixture parameter used to balance the contributions of the two exponential functions. The mixed exponential loss function highlights the importance of samples involved in the target class while reducing the influence of samples that are outliers. This approach significantly enhances the robustness of the SVDD model. This loss function achieves more accurate and stable anomaly detection results in various settings by preserving the integrity of the target class and diminishing the effect of potential anomalies.

## 3. Truncated Loss Function

SVDD models with unbounded loss functions can achieve satisfactory results when addressing scenarios lacking noise. However, the continual growth of these loss functions results in the collapse of the model when it is subjected to noise. Therefore, truncating the SVDD model’s loss function makes it more robust. The general definition of a truncated loss function is as follows:

(15) $$L\left(u,\delta \right)=\phi \left(u\right)-{\left(\phi \left(u\right)-\delta \right)}_{+}$$

where δ is a constant, and ϕ(u) is an unbounded loss function, such that, when u≤0, ϕ(u)=0. Since ϕ(u) is an abstract function, a general form of the truncated loss function includes several loss functions. The three specific truncated loss functions we created in our study are present as follows:

1. Truncated generalized ramp loss function: $L_{\phi_{R}}\left(u,\delta \right)=\min\left\{0,\max\left(\delta ,\phi_{R}\left(u\right)\right)\right\}$, where $\phi_{R}\left(u\right)=\frac{u}{v}$, u,v>0.
2. Truncated binary cross entropy loss function: $L_{\phi_{f}}\left(u,\delta \right)=\min\left\{0,\max\left(\delta ,\phi_{f}\left(u\right)\right)\right\}$, where $\phi_{f}\left(u\right)=\log\left(1+\frac{u}{\theta }\right)$, u,θ>0.
3. Truncated linear exponential loss function: $L_{\phi_{L}}\left(u,\delta \right)=\min\left\{0,\max\left(\delta ,\phi_{L}\left(u\right)\right)\right\}$, where $\phi_{L}\left(u\right)=\exp\left(ayu\right)-ayu-1$, u,a>0, and y=±1.

Assuming the truncation point $u=\delta^{\prime }$, the mathematical properties of the three truncated loss functions presented above can be summarized as follows:

1. For samples with u≤0, the loss value is 0; for samples with $u>\delta^{\prime }$, the loss value is δ. Thus, the general truncated loss function exhibits sparsity and robustness to outliers.
2. $L_{\phi_{R}}\left(u,\delta \right)$ and $L_{\phi_{M}}\left(u,\delta \right)$ are truncated concave loss functions, which are non-differentiable at $u=0,\delta^{\prime }$. $L_{\phi_{L}}\left(u,\delta \right)$ is a truncated convex loss function, which is non-differentiable at $u=\delta^{\prime }$ and differentiable at u=0.
3. $L_{\phi_{R}}\left(u,\delta \right)$ and $L_{\phi_{f}}\left(u,\delta \right)$ exhibit explicit expressions for the proximal operators, while $L_{\phi_{L}}\left(u,\delta \right)$ does not.

In the next section, we provide explicit expressions for the proximal operators of $L_{\phi_{R}}\left(u,\delta \right)$ and $L_{\phi_{f}}\left(u,\delta \right)$.

### 3.1. Proximal Operators of Truncated Loss Functions

**Definition 1**  (Proximal Operator [35])**.** *Assume* f*: $\mathbb{R}\to \bar{\mathbb{R}}$* *is a proper lower-semi-continuous loss function. The expression for the proximal operator of f(u)* *at x∈ℝ* *is defined as follows:*

(16) $$prox_{\lambda f}\left(x\right)=\arg\min\left\{f\left(u\right)+\frac{1}{2\lambda }{\left\|u-x\right\|}^{2}\right\}$$

*when* f(u) *is a convex loss function, it presents a single-value proximal operator; when* f(u) *is a non-convex loss function, it exhibits a multi-value proximal operator.*

**Lemma 1**  ([36])**.** *When $f\left(u\right)=\log\left(1+\frac{u}{\theta }\right)$* *, let $v\left(x\right)=\arg\underset{u\in \mathbb{R}}{\min}\left\{\log\left(1+\frac{u}{\theta }\right)+\frac{1}{2\lambda }{\left\|u-x\right\|}^{2}\right\}$* *,* x∈ℝ*. The expressions for the proximal operators are as follows:*

(17) $$v\left(x\right)=\{\begin{array}{cc} \bar{v}\left(x\right) & {\left(x-\theta \right)}^{2}-4\left(\lambda -x\theta \right)\geq 0\;and\;u>0\\ 0 & othersize \end{array}$$

$\bar{v}\left(x\right)=\min\left\{0,{\left[\frac{\left(x-\theta \right)+\sqrt{{\left(x-\theta \right)}^{2}-4\left(\lambda -x\theta \right)}}{2}\right]}_{+},{\left[\frac{\left(x-\theta \right)-\sqrt{{\left(x-\theta \right)}^{2}-4\left(\lambda -x\theta \right)}}{2}\right]}_{+}\right\}$.

**Lemma 2.**  *The explicit expression of the* $L_{\phi_{R}}\left(u,\delta \right)$ *proximal operator is as follows:*

*1.*  *When* $0<\lambda <2\delta v^{2}$ *, the explicit expression of the* $L_{\phi_{R}}\left(u,\delta \right)$ *proximal operator is presented as follows:*
(18) $$prox_{\lambda L_{R}}\left(x\right)=\{\begin{array}{cc} x & x>\delta v+\frac{\lambda }{2v}\\ \left\{x,x-\frac{\lambda }{v}\right\} & x=\delta v+\frac{\lambda }{2v}\\ x-\frac{\lambda }{v} & \frac{\lambda }{v}<x<\delta v+\frac{\lambda }{2v}\\ 0 & 0\leq x\leq \frac{\lambda }{v}\\ x & x<0 \end{array}$$
*2.*  *When* $0<\lambda <2\delta v^{2}$ *, the explicit expression of the* $L_{\phi_{R}}\left(u,\delta \right)$ *proximal operator is as follows: *
(19) $$prox_{\lambda L_{R}}\left(x\right)=\{\begin{array}{cc} x & x>\sqrt{2\lambda \delta }\\ \left\{x,0\right\} & x=\sqrt{2\lambda \delta }\\ 0 & 0\leq x\leq \sqrt{2\lambda \delta }\\ x & x<0 \end{array}$$

**Proof of Lemma 1.**  Equation (16) exhibits that $prox_{L_{\phi_{R}}}$ is a local minimum of the following piecewise function:

$$\varphi \left(u\right)=\{\begin{array}{cc} \varphi_{1}\left(u\right)=\delta +\frac{1}{2\lambda }{\left(u-x\right)}^{2} & u>\delta v\\ \varphi_{2}\left(u\right)=\delta +\frac{1}{2\lambda }{\left(1-x\right)}^{2} & u=\delta v\\ \varphi_{3}\left(u\right)=\frac{u}{v}+\frac{1}{2\lambda }{\left(u-x\right)}^{2} & 0<u<\delta v\\ \varphi_{4}\left(u\right)=\frac{x^{2}}{2\lambda } & u=0\\ \varphi_{5}\left(u\right)=\frac{1}{2\lambda }{\left(u-x\right)}^{2} & u<0 \end{array}$$

The minima of the piecewise functions $\varphi_{1}\left(u\right)$, $\varphi_{2}\left(u\right)$, $\varphi_{3}\left(u\right)$, $\varphi_{4}\left(u\right)$, and $\varphi_{5}\left(u\right)$ are located at $u_{1}^{*}=x$, $u_{2}^{*}=1$, $u_{3}^{*}=x-\frac{\lambda }{v}$, $u_{4}^{*}=0$, and $u_{5}^{*}=x$, with minimum values of $\varphi_{1}\left(u_{1}^{*}\right)=\delta$, $\varphi_{2}\left(u_{2}^{*}\right)=\delta +\frac{1}{2\lambda }{\left(\delta v-x\right)}^{2}$, $\varphi_{3}\left(u_{3}^{*}\right)=\frac{x}{v}-\frac{\lambda }{2v^{2}}$, $\varphi_{4}\left(u_{4}^{*}\right)=\frac{x^{2}}{2\lambda }$, and $\varphi_{5}\left(u_{5}^{*}\right)=0$, respectively.

Since $0<u_{3}^{*}=x-\frac{\lambda }{v}<v\delta$, it follows that $\frac{\lambda }{v}<x<v\delta +\frac{\lambda }{v}$. If $\varphi_{1}\left(u_{1}^{*}\right)<\varphi_{3}\left(u_{3}^{*}\right)$, then $x>\delta v+\frac{\lambda }{2v}$. When $\delta v+\frac{\lambda }{2v}\leq \frac{\lambda }{v}$ and x is in the interval $\left(\frac{\lambda }{v},v\delta +\frac{\lambda }{v}\right)$, $\varphi_{1}\left(u_{1}^{*}\right)<\varphi_{3}\left(u_{3}^{*}\right)$.

When $0<\lambda <2\delta v^{2}$, the following conclusion can be reached by comparing the values of $\varphi_{1}\left(u_{1}^{*}\right)$, $\varphi_{2}\left(u_{2}^{*}\right)$, $\varphi_{3}\left(u_{3}^{*}\right)$, $\varphi_{4}\left(u_{4}^{*}\right)$, and $\varphi_{5}\left(u_{5}^{*}\right)$.

(1.1) Since $x>v\delta +\frac{\lambda }{2v}$, we achieve $\min\left\{\varphi_{2}\left(u_{2}^{*}\right),\varphi_{3}\left(u_{3}^{*}\right),\varphi_{4}\left(u_{4}^{*}\right)\right\}>\varphi_{1}\left(u_{1}^{*}\right)$, which means $u^{*}=u_{1}^{*}=x$.
(1.2) Since $x=v\delta +\frac{\lambda }{2v}$, we obtain $\min\left\{\varphi_{2}\left(u_{2}^{*}\right),\varphi_{4}\left(u_{4}^{*}\right)\right\}>\varphi_{1}\left(u_{1}^{*}\right)=\varphi_{3}\left(u_{3}^{*}\right)$, which means $u^{*}=u_{1}^{*}=x$ or $u^{*}=u_{3}^{*}=x-\frac{\lambda }{v}$.
(1.3) Since $\frac{\lambda }{v}<x<v\delta +\frac{\lambda }{2v}$, we achieve $\min\left\{\varphi_{1}\left(u_{1}^{*}\right),\varphi_{2}\left(u_{2}^{*}\right),\varphi_{4}\left(u_{4}^{*}\right)\right\}>\varphi_{3}\left(u_{3}^{*}\right)$, which means $u^{*}=u_{3}^{*}=x-\frac{\lambda }{v}$.
(1.4) Since $0\leq x\leq \frac{\lambda }{v}$, we obtain $\min\left\{\varphi_{1}\left(u_{1}^{*}\right),\varphi_{2}\left(u_{3}^{*}\right),\varphi_{3}\left(u_{3}^{*}\right)\right\}>\varphi_{4}\left(u_{4}^{*}\right)$, which means $u^{*}=u_{4}^{*}=0$.
(1.5) Since x<0, we achieve $\min\left\{\varphi_{2}\left(u_{2}^{*}\right),\varphi_{4}\left(u_{4}^{*}\right)\right\}>\varphi_{5}\left(u_{5}^{*}\right)$, which means $u^{*}=u_{5}^{*}=x$.

According to (1.1)–(1.5), Equation (18) can be derived.

When $\lambda \geq 2\delta v^{2}$, the following conclusion can be reached by comparing the values of $\varphi_{1}\left(u_{1}^{*}\right)$, $\varphi_{2}\left(u_{2}^{*}\right)$, $\varphi_{3}\left(u_{3}^{*}\right)$, $\varphi_{4}\left(u_{4}^{*}\right)$, and $\varphi_{5}\left(u_{5}^{*}\right)$.

(2.1) As $x>\sqrt{2\lambda \delta }$, we obtain $\min\left\{\varphi_{2}\left(u_{2}^{*}\right),\varphi_{3}\left(u_{3}^{*}\right),\varphi_{4}\left(u_{4}^{*}\right)\right\}>\varphi_{1}\left(u_{1}^{*}\right)$, which means $u^{*}=u_{1}^{*}=x$.
(2.2) As $x=\sqrt{2\lambda \delta }$, we obtain $\min\left\{\varphi_{2}\left(u_{2}^{*}\right),\varphi_{3}\left(u_{3}^{*}\right)\right\}>\varphi_{1}\left(u_{1}^{*}\right)=\varphi_{4}\left(u_{4}^{*}\right)$, which either means $u^{*}=u_{1}^{*}=x$ or $u^{*}=u_{4}^{*}=0$.
(2.3) As $0\leq x<\sqrt{2\lambda \delta }$, we obtain $\min\left\{\varphi_{1}\left(u_{1}^{*}\right),\varphi_{2}\left(u_{3}^{*}\right),\varphi_{3}\left(u_{3}^{*}\right)\right\}>\varphi_{4}\left(u_{4}^{*}\right)$, which means $u^{*}=u_{4}^{*}=0$.
(2.4) As x<0, we obtain $\min\left\{\varphi_{1}\left(u_{1}^{*}\right),\varphi_{2}\left(u_{2}^{*}\right),\varphi_{3}\left(u_{3}^{*}\right),\varphi_{4}\left(u_{4}^{*}\right)\right\}>\varphi_{5}\left(u_{5}^{*}\right)$, which means $u^{*}=u_{5}^{*}=x$.

According to (2.1)–(2.4), Equation (19) can be derived. □

**Lemma 3.**  *The expression representing the proximal operator of the truncation function is as follows:*

(20) $$prox_{\lambda L}\left(x\right)=\{\begin{array}{cc} x & x>\lambda^{\prime }\;and\;\varphi_{1}\left(u_{1}^{*}\right)<\varphi_{3}\left(u_{3}^{*}\right)\;and\;\varphi_{1}\left(u_{1}^{*}\right)<\varphi_{4}\left(u_{4}^{*}\right)\\ \left\{x,v\left(x\right)\right\} & x=\lambda^{\prime }\;and\;\varphi_{1}\left(u_{1}^{*}\right)=\varphi_{3}\left(u_{3}^{*}\right)<\varphi_{4}\left(u_{4}^{*}\right)\\ \left\{x,0\right\} & x=\lambda^{\prime }\;and\;\varphi_{1}\left(u_{1}^{*}\right)=\varphi_{4}\left(u_{4}^{*}\right)<\varphi_{3}\left(u_{3}^{*}\right)\\ v\left(x\right) & \omega_{down}<x<\omega_{up}\;and\;\varphi_{1}\left(u_{1}^{*}\right)>\varphi_{3}\left(u_{3}^{*}\right)\;and\;\varphi_{3}\left(u_{3}^{*}\right)<\varphi_{4}\left(u_{4}^{*}\right)\\ 0 & x\geq 0\;and\;\varphi_{3}\left(u_{3}^{*}\right)>\varphi_{4}\left(u_{4}^{*}\right)\;and\;\varphi_{1}\left(u_{1}^{*}\right)>\varphi_{4}\left(u_{4}^{*}\right)\\ x & x<0 \end{array}$$

*where* $u_{1}^{*}$ *,* $u_{2}^{*}$ *,* $u_{3}^{*}$ *, and* $u_{4}^{*}$ *represent the minimizers of the piecewise function, and* $\varphi_{1}\left(u_{1}^{*}\right)$ *,* $\varphi_{2}\left(u_{2}^{*}\right)$ *,* $\varphi_{3}\left(u_{3}^{*}\right)$ *, and* $\varphi_{4}\left(u_{4}^{*}\right)$ *, represent the minimal values of the piecewise function.*

**Proof of Lemma 3.**  It can be deduced from Equations (15) and (16) that $prox_{\lambda L}$ represents the local minimum of the following piecewise function:

$$\varphi \left(u\right)=\{\begin{array}{cc} \varphi_{1}\left(u\right)=\delta +\frac{1}{2\lambda }{\left(u-x\right)}^{2} & u>\lambda^{\prime }\\ \varphi_{2}\left(u\right)=\delta +\frac{1}{2\lambda }{\left(\lambda^{\prime }-x\right)}^{2} & u=\lambda^{\prime }\\ \varphi_{3}\left(u\right)=\phi \left(u\right)+\frac{1}{2\lambda }{\left(u-x\right)}^{2} & 0<u<\lambda^{\prime }\\ \varphi_{4}\left(u\right)=\frac{x^{2}}{2\lambda } & u=0\\ \varphi_{5}\left(u\right)=\frac{1}{2\lambda }{\left(u-x\right)}^{2} & x<0 \end{array}$$

Let $v\left(x\right)=\arg\min\phi \left(u\right)+\frac{1}{2\lambda }{\left(u-x\right)}^{2}$; the minimizers of the piecewise functions are $u_{1}^{*}=x$, $u_{2}^{*}=\lambda^{\prime }$, $u_{3}^{*}=v\left(x\right)$, $u_{4}^{*}=0$, and $u_{5}^{*}=x$, and their minimal values are $\varphi_{1}\left(u_{1}^{*}\right)=\delta$, $\varphi_{2}\left(u_{2}^{*}\right)=\delta +\frac{1}{2\lambda }{\left(\lambda^{\prime }-x\right)}^{2}$, $\varphi_{3}\left(u_{3}^{*}\right)=\varphi_{3}\left(v\left(x\right)\right)$, $\varphi_{4}\left(u_{4}^{*}\right)=\frac{x^{2}}{2\lambda }$, and $\varphi_{5}\left(u_{5}^{*}\right)=0$, respectively. From $0<u_{3}^{*}=v\left(x\right)<\lambda^{\prime }$, it follows that $\omega_{down}<x<\omega_{up}$.

When x≥0, the stage function’s minimal values are $\varphi_{1}\left(u_{1}^{*}\right)$,$\varphi_{2}\left(u_{2}^{*}\right)$,$\varphi_{3}\left(u_{3}^{*}\right)$, and $\varphi_{4}\left(u_{4}^{*}\right)$; when x<0, the stage function’s minimal values are $\varphi_{2}\left(u_{2}^{*}\right)$, $\varphi_{4}\left(u_{4}^{*}\right)$, and $\varphi_{5}\left(u_{5}^{*}\right)$. Thus, we can observe that $\varphi_{2}\left(u_{2}^{*}\right)\geq \varphi_{1}\left(u_{1}^{*}\right)$. If $\varphi_{1}\left(u_{1}^{*}\right)\geq \varphi_{3}\left(u_{3}^{*}\right)$, then $\varphi_{2}\left(u_{2}^{*}\right)\geq \varphi_{1}\left(u_{1}^{*}\right)\geq \varphi_{3}\left(u_{3}^{*}\right)$; similarly, if $\varphi_{1}\left(u_{1}^{*}\right)\geq \varphi_{4}\left(u_{4}^{*}\right)$, then $\varphi_{2}\left(u_{2}^{*}\right)\geq \varphi_{1}\left(u_{1}^{*}\right)\geq \varphi_{4}\left(u_{4}^{*}\right)$. We can determine the following conclusions by comparing the values of $\varphi_{1}\left(u_{1}^{*}\right)$, $\varphi_{2}\left(u_{2}^{*}\right)$, $\varphi_{3}\left(u_{3}^{*}\right)$, $\varphi_{4}\left(u_{4}^{*}\right)$, and $\varphi_{5}\left(u_{5}^{*}\right)$:

(1.1) When the conditions of $x>\lambda^{\prime }$, $\varphi_{1}\left(u_{1}^{*}\right)<\varphi_{3}\left(u_{3}^{*}\right)$, and $\varphi_{1}\left(u_{1}^{*}\right)<\varphi_{4}\left(u_{4}^{*}\right)$ are met, and it follows that $u^{*}=u_{1}^{*}=x$;
(1.2) When the condition of $x=\lambda^{\prime }$ is met, if $\varphi_{1}\left(u_{1}^{*}\right)=\varphi_{3}\left(u_{3}^{*}\right)<\varphi_{4}\left(u_{4}^{*}\right)$ is true, then $u^{*}=u_{1}^{*}=x$ or $u^{*}=u_{3}^{*}=v\left(x\right)$ can be derived;
(1.3) When the condition of $x=\lambda^{\prime }$ is met, if $\varphi_{1}\left(u_{1}^{*}\right)=\varphi_{4}\left(u_{4}^{*}\right)<\varphi_{3}\left(u_{3}^{*}\right)$ is true, then $u^{*}=u_{1}^{*}=x$ or $u^{*}=u_{4}^{*}=0$ can be derived;
(1.4) When the conditions of $\omega_{down}<x<\omega_{up}$, $\varphi_{1}\left(u_{1}^{*}\right)>\varphi_{3}\left(u_{3}^{*}\right)$, and $\varphi_{3}\left(u_{3}^{*}\right)<\varphi_{4}\left(u_{4}^{*}\right)$ are met, it follows that $u^{*}=u_{3}^{*}=v\left(x\right)$;
(1.5) When the conditions of x≥0, $\varphi_{1}\left(u_{1}^{*}\right)>\varphi_{4}\left(u_{4}^{*}\right)$, and $\varphi_{4}\left(u_{4}^{*}\right)<\varphi_{3}\left(u_{3}^{*}\right)$ are met, it follows that $u^{*}=u_{4}^{*}=0$;
(1.6) When the condition of x<0 is met, it follows that $u^{*}=u_{5}^{*}=x$.

Thus, it is possible to successfully derive Equation (20). □

### 3.2. The Use of the Proximal Operator Algorithm to Solve Truncated Loss Functions

When ϕ(u) in the truncated loss function is a monotonic and non-piecewise function, and $v\left(x\right)=\arg\min\phi \left(u\right)+\frac{1}{2\lambda }{\left(u-x\right)}^{2}$ can be expressed explicitly, the proximal operator of the truncated loss function can be calculated using Formula (20). In practical applications, however, it is sometimes impossible to obtain the explicit expression for v(x); for example, $\phi_{L}\left(u\right)=\exp\left(ayu\right)-ayu-1$ does not provide an explicit expression. The calculation of the proximal operator in such scenarios is discussed below.

For $\arg\min\phi \left(u\right)+\frac{1}{2\lambda }{\left(u-x\right)}^{2}$, if it is smooth and has a second derivative, the problem is a smooth unconstrained optimization problem. Newton’s method is used to solve for the minimum of φ(u) in unconstrained optimization problems due to its high convergence rate. The gradient and Hessian matrix for problem (16) can be expressed as follows:

(21) $$g=\frac{\partial \phi \left(u\right)}{\partial u}+\frac{1}{\lambda }\left(u-x\right)$$

(22) $$H=\frac{\partial^{2}\phi \left(u\right)}{\partial u^{2}}+\frac{1}{\lambda }I$$

The minimizer $u_{3}^{*}$ and the minimal value $\varphi_{3}\left(u_{3}^{*}\right)$ can be obtained with Newton’s method for x.

If an explicit expression for v(x) cannot be achieved, the calculation of the proximal operator follows the same process as Lemma 3. Once the minimizers $u_{3}^{*}$ and $\varphi_{3}\left(u_{3}^{*}\right)$ are obtained, the proximal operator can be calculated. When the conditions of $0<u_{3}^{*}<\lambda^{\prime }$, $\varphi_{1}\left(u_{1}^{*}\right)>\varphi_{3}\left(u_{3}^{*}\right)$, and $\varphi_{3}\left(u_{3}^{*}\right)<\varphi_{4}\left(u_{4}^{*}\right)$ are met, we can derive $u^{*}=u_{3}^{*}$. Therefore, Formula (20) can be modified to express the following:

(23) $$prox_{\lambda L}\left(x\right)=\{\begin{array}{cc} x & x>\lambda^{\prime }\;and\;\varphi_{1}\left(u_{1}^{*}\right)<\varphi_{3}\left(u_{3}^{*}\right)\;and\;\varphi_{1}\left(u_{1}^{*}\right)<\varphi_{4}\left(u_{4}^{*}\right)\\ \left\{x,u_{3}^{*}\right\} & x=\lambda^{\prime }\;and\;\varphi_{1}\left(u_{1}^{*}\right)=\varphi_{3}\left(u_{3}^{*}\right)<\varphi_{4}\left(u_{4}^{*}\right)\\ \left\{x,0\right\} & x=\lambda^{\prime }\;and\;\varphi_{1}\left(u_{1}^{*}\right)=\varphi_{4}\left(u_{4}^{*}\right)<\varphi_{3}\left(u_{3}^{*}\right)\\ u_{3}^{*} & 0<u_{3}^{*}<\lambda^{\prime }\;and\;\varphi_{1}\left(u_{1}^{*}\right)>\varphi_{3}\left(u_{3}^{*}\right)\;and\;\varphi_{3}\left(u_{3}^{*}\right)<\varphi_{4}\left(u_{4}^{*}\right)\\ 0 & x\geq 0\;and\;\varphi_{3}\left(u_{3}^{*}\right)>\varphi_{4}\left(u_{4}^{*}\right)\;and\;\varphi_{1}\left(u_{1}^{*}\right)>\varphi_{4}\left(u_{4}^{*}\right)\\ x & x<0 \end{array}$$

Based on the analysis presented above, the algorithm for solving the proximal operator of the truncated loss function is as following Algorithm 1:
**Algorithm 1:** Algorithm for solving the proximal operator of the truncated loss functionInput: x,δ,λ, ϕ(u)$\mathrm{Output}:\;\mathrm{Proximal}\;\mathrm{operator}\;u^{*}$$1:\;\mathrm{Choose}\;\mathrm{an}\;\mathrm{initial}\;\mathrm{point}\;u^{\left(0\right)}=x$.2: While t≤MAX do $3:\;\mathrm{Calculate}\;g^{\left(t\right)}$ according to Formula (21). $4:\;\mathrm{If}\;\left\|g^{\left(t\right)}\right\|<eps$$,\;\mathrm{then}\;\mathrm{stop}\;\mathrm{the}\;\mathrm{loop},\;\mathrm{the}\;\mathrm{approximate}\;\mathrm{solution}\;u_{3}^{*}=u^{\left(t+1\right)}$ is obtained.$5:\;\;\;\mathrm{Calculate}\;H^{\left(t\right)}$ according to Formula (22).$6:\;\;\;\mathrm{Calculate}\;\bar{d},\;\mathrm{the}\;\mathrm{Newton}\;\mathrm{iteration}\;\mathrm{direction},\;\mathrm{according}\;\mathrm{to}\;\mathrm{the}\;\mathrm{Newton}\;\mathrm{iteration}\;\mathrm{equation}\;H^{\left(t\right)}d=-g^{\left(t\right)}$.$7:\;\;\;\mathrm{Solve}\;\mathrm{for}\;\mathrm{the}\;\mathrm{step}\;\mathrm{size}\;\theta \;\mathrm{using}\;\mathrm{the}\;\mathrm{backtracking}\;\mathrm{Armijo}\;\mathrm{method},\;\mathrm{and}\;\mathrm{update}\;u^{\left(t\right)}=u^{\left(t-1\right)}+\theta \bar{d}$. 8:  Set t=t+1.9:  End$10:\mathrm{Calculate}\;\varphi \left(u_{3}^{*}\right)\;\mathrm{according}\;\mathrm{to}\;u_{3}^{*}$.$11:\mathrm{Calculate}\;\mathrm{the}\;\mathrm{proximal}\;\mathrm{operator}\;u^{*}$ according to Formula (23).

## 4. Robust SVDD Model

Formula (1), for the SVDD formula, can be rewritten as follows:

(24) $$\underset{R,\mu ,\xi_{i}}{\min}\;R^{2}+C\sum_{i}{\left[u_{i}\right]}_{+},u_{i}={\left\|\varphi \left(x_{i}\right)-\mu \right\|}^{2}-R^{2}$$

where ${\left[u\right]}_{+}=\max(0,u)$ represents the hinge loss function. Since the hinge loss function is sensitive to outliers, it can be replaced with the truncated loss function from Formula (15). Thus, the objective function for obtaining the robust SVDD model is as follows:

(25) $$\underset{R,\mu ,\xi_{i}}{\min}\;R^{2}+C\sum_{i}\left(\phi \left(u\right)-{\left(\delta -\phi \left(u\right)\right)}_{+}\right),u_{i}={\left\|\varphi \left(x_{i}\right)-\mu \right\|}^{2}-R^{2}$$

As the truncated loss function is non-differentiable, solving the objective function of the robust SVDD model is a non-convex optimization problem, and it cannot be solved using standard SVDD model methods.

**Theorem 1**  (Nonparametric Representation Theorem [37])**.** *Suppose we are designated a non-empty set χ* *; a positive definite real-valued kernel* *K: *χ×χ→ℝ*; a training sample* $\left(x_{i},y_{i}\right)\left(i\in {\mathbb{N}}_{m}\right)\in \chi \times \mathbb{R}$*; a strictly monotonically increasing real-valued function* g* on *[0,+∞]*; an arbitrary cost function* c*: *${\left(\chi \times {\mathbb{R}}^{2}\right)}^{m}\to \mathbb{R}\cup \left\{\infty \right\}$*; and a class of functions: *

$$F=\left\{f\in {\mathbb{R}}^{\chi }|f\left(\cdot \right)=\sum_{i=1}^{m}\beta_{i}K\left(z_{i},\cdot \right),\beta_{i}\in \mathbb{R},z_{i}\in \chi ,\left\|f\right\|\leq \infty \right\}$$

In this scenario, ‖⋅‖ represents the norm in RKHS, $H_{k}$ associated with K(⋅,⋅), i.e., for any $z_{i}\in \chi$.

(26) $${\left\|\sum_{i=1}^{\infty }\beta_{i}K\left(z_{i},\cdot \right)\right\|}^{2}=\sum_{i=1}^{\infty }\sum_{j=1}^{\infty }\beta_{i}\beta_{j}K\left(z_{i},z_{j}\right)$$

Then, any f∈F minimizing the regularized risk function

(27) $$c\left(\left(x_{1},x_{1},y_{1},f\left(x_{1}\right)\right),\cdots ,\left(x_{m},y_{m},f\left(x_{m}\right)\right)\right)+g\left(\left\|f\right\|\right)$$

admits a representation of $f\left(\cdot \right)=-\sum_{i=1}^{m}a_{i}y_{i}K\left(x_{i},\cdot \right)$, where $a_{i}\in \mathbb{R}\left(i\in {\mathbb{N}}_{m}\right)$ represents coefficients of f in RKHS $H_{k}$.

A set of vectors a exists in the nonparametric representation theorem, where the center $\mu =\sum_{i=1}^{n}a_{i}\phi \left(x_{i}\right)$ is the optimal solution for problem (25). Therefore, Formula (25) can be transformed into the following:

(28) $$\begin{array}{l} \underset{R,u_{i}}{\min}\;R^{2}+C\sum_{i}L\left(u_{i}\right)\\ u_{i}=K\left(x_{i},x_{i}\right)-2\sum_{j=1}^{n}a_{j}K\left(x_{i},x_{j}\right)+\sum_{j=1}^{n}\sum_{k=1}^{n}a_{j}a_{k}K\left(x_{j},x_{k}\right)-R^{2} \end{array}$$

Formula (28) represents the single-class SVDD model. To obtain data that include negative samples, these samples must be integrated into the SVDD model; then, the center is $\mu =\sum_{i=1}^{n}a_{i}y_{i}\phi \left(x_{i}\right)$, and the objective function of the robust SVDD model is as follows:

(29) $$\begin{array}{l} \underset{R,\mu ,\xi_{i}}{\min}\;R^{2}+\sum_{i}C_{y_{i}}L\left(u_{i}\right)\\ u_{i}=y_{i}K\left(x_{i},x_{i}\right)-2y_{i}\sum_{j=1}^{n}a_{j}y_{j}K\left(x_{i},x_{j}\right)+y_{i}\sum_{j=1}^{n}\sum_{k=1}^{n}a_{j}y_{j}a_{k}y_{k}K\left(x_{j},x_{k}\right)-y_{i}R^{2} \end{array}$$

when $a_{y}=a.*y$, it follows that $\mu =\sum_{i=1}^{n}a_{y_{i}}\phi \left(x_{i}\right)$. Problem (29) is rewritten as the following matrix form:

(30) $$\begin{array}{l} \underset{R,u}{\min}\;R^{2}+CL_{\phi }\left(u\right)\\ u=K_{a}-2K_{b}a_{y}+a_{y}^{T}Ka_{y}D-R^{2}D,R^{2}>0 \end{array}$$

where $K_{a}=diag(K).*y$, $K_{b}=\left[y_{1}*K\left(1,\cdot \right),y_{2}*K\left(2,\cdot \right),\cdots ,y_{n}*K\left(n,\cdot \right)\right]$, $D={\left[y_{1},y_{2}\cdots ,y_{n}\right]}^{T}$, and $C=\left[C_{y_{1}},C_{y_{2}}\cdots ,C_{y_{n}}\right]$. This study discusses the use of the SVDD model as a solution for addressing data with negative samples, and the Lagrangian function for problem (30) is as follows:

(31) $$L_{\beta }\left(R^{2},a_{y},u,\eta ,\beta \right)=R^{2}+CL_{\phi }\left(u\right)+\langle \eta ,K_{a}-2K_{b}a_{y}+a_{y}^{T}Ka_{y}D-R^{2}D-u\rangle$$

The KKT conditions for problem (30) are provided below:

(32) $$\{\begin{array}{c} K_{a}-2K_{b}a_{y}^{*}+a_{y}^{{*}^{T}}Ka_{y}^{*}D-R^{2^{*}}D-u^{*}=0\\ 0=1-\eta^{{*}^{T}}D\\ 0=\left(2D{a_{y}^{*}}^{T}K-2{K_{b}}^{T}\right)\eta^{{*}^{T}}\\ 0\in \partial CL_{\phi }\left(u^{*}\right)-\eta^{*} \end{array}$$

where $\left(R^{2^{*}},a_{y}^{*},u^{*}\right)$ represents any KKT point.

The generalized non-smooth optimization problem can be represented by the following Formula [38]:

(33) $$\begin{array}{l} \underset{R,u}{\min}\;f\left(s\right)+cl\left(q\right)\\ q+g\left(s\right)=0 \end{array}$$

where f(⋅) and g(⋅) are continuously differentiable functions on ${\mathbb{R}}^{n}\to \mathbb{R}$, and l(q) represents a non-smooth function on ${\mathbb{R}}^{n}\to \mathbb{R}$. Problem (31) is considered as a form of the aforementioned generalized non-smooth optimization problem, where $s=\left[R^{2},a_{y}\right]$, q=u, $f\left(s\right)=s_{1}$, and $g\left(s\right)=-K_{a}+2K_{b}s_{2}-s_{2}^{T}Ks_{2}D+s_{1}D$, representing the optimization model’s constraints, are nonlinear equality constraints. In this study, the fast ADMM algorithm was employed to solve problem (33), and the algorithm will be introduced in a subsequent chapter.

## 5. Fast ADMM Algorithm

The previous section presented the optimization mathematical model of the robust SVDD model. Since the optimization mathematical model includes nonlinear equality constraints, the fast ADMM method was used to solve Formula (33). The augmented Lagrangian function of the robust SVDD model is as follows:

(34) $$L_{\beta }\left(R^{2},a_{y},u,\eta ,\beta \right)=R^{2}+CL_{\phi }\left(u\right)+\langle \eta ,g\left(a_{y}\right)-R^{2}D-u\rangle +\frac{\beta }{2}{\left\|g\left(a_{y}\right)-R^{2}D-u\right\|}^{2}$$

where $\lambda \in {\mathbb{R}}^{m}$ represents the vector of Lagrange multipliers, and β>0 represents the penalty factor, $g\left(a_{y}\right)=K_{a}-2K_{b}a_{y}+a_{y}^{T}Ka_{y}D$.

### 5.1. Fast ADMM Algorithm Framework

Based on the augmented Lagrangian function previously presented, we obtained the following framework for the fast ADMM algorithm:

(35) $$u^{k+1}=\underset{u\in {\mathbb{R}}^{m}}{\arg\min}L_{\beta }\left(R^{2^{k}},a_{y}^{k},u,\eta^{k}\right)$$

(36) $$a_{y}^{k+1}=\underset{a_{y}\in {\mathbb{R}}^{m}}{\arg\min}L_{\beta }\left(R^{2^{k}},a_{y},u^{k+1},\eta^{k}\right)+\Delta \varphi_{1}\left(a_{y},a_{y}^{k}\right)$$

(37) $$R^{2k+1}=\underset{R^{2}\in {\mathbb{R}}^{m}}{\arg\min}L_{\beta }\left(R^{2},a_{y}^{k+1},u^{k+1},\eta^{k}\right)+\Delta \varphi_{2}\left(R^{2},R^{2^{k}}\right)$$

(38) $$\eta^{k+1}=\eta^{k}+\beta \left(g\left(a_{y}^{k+1}\right)-R^{2^{k+1}}D-u^{k+1}\right)$$

**1.**  **Computing** $u^{k+1}$

The solution of $u^{k+1}$ in Formula (35) is equivalent to the following problem:

(39) $$\begin{array}{l} u^{k+1}=\underset{u\in {\mathbb{R}}^{m}}{\arg\min}CL_{\phi }\left(u\right)+\frac{\beta }{2}{\left\|\left(g\left(a_{y}^{k}\right)-R^{2^{k}}D+\frac{\eta^{k}}{\beta }\right)-u\right\|}^{2}\\ =\underset{u\in {\mathbb{R}}^{m}}{\arg\min}CL_{\phi }\left(u\right)+\frac{\beta }{2}{\left\|z^{k}-u\right\|}^{2}\\ =prox_{\frac{c}{\beta }L_{\phi }}\left(z^{k}\right) \end{array}$$

where $z^{k}=g\left(a_{y}^{k}\right)-R^{2^{k}}D+\frac{\eta^{k}}{\beta }$. Therefore, the calculation of $u^{k+1}$ can be transformed into the computation of the proximal operator, which can be determined using Formula (20) or Algorithm 1.

**2.**  **Computing** $a_{y}^{k+1}$

When solving for variable $a_{y}^{k+1}$, if a closed-form solution for this subproblem is not obtained, an optimization algorithm must be used for the iterative solution, which results in a slow computational speed. Thus, to solve this problem in a more efficient manner, we used a linearization technique.

Given the convex differentiable function φ, the Bregman distance between x and y,x,y∈dom(φ) is defined as follows:

(40) Δφ(x,y)=φ(x)−φ(y)−(∇φ(y),x−y)

When $\varphi \left(x\right)=\frac{\mu }{2}{\left\|x\right\|}^{2}-\frac{\beta }{2}{\left\|g\left(x\right)-R^{2^{k}}D-u^{k+1}+\frac{\eta^{k}}{\beta }\right\|}^{2}$, the Bregman distance Δφ(x,y) is as follows:

(41) $$\begin{array}{c} \Delta \varphi \left(x,y\right)=\frac{\mu }{2}{\left\|x-y\right\|}^{2}-\frac{\beta }{2}{\left\|g\left(x\right)-R^{2^{k}}D-u^{k+1}+\frac{\eta^{k}}{\beta }\right\|}^{2}+\frac{\beta }{2}{\left\|g\left(y\right)-R^{2^{k}}D-u^{k+1}+\frac{\eta^{k}}{\beta }\right\|}^{2}\\ +\langle \beta \nabla g\left(y\right)\left(g\left(y\right)-R^{2^{k}}D-u^{k+1}+\frac{\eta^{k}}{\beta }\right),x-y\rangle \end{array}$$

Formula (36) is equivalent to the following:

(42) $$\begin{array}{l} a_{y}^{k+1}=\underset{a_{y}\in {\mathbb{R}}^{m}}{\arg\min}\frac{\mu }{2}{\left\|a_{y}-a_{y}^{k}\right\|}^{2}+\frac{\beta }{2}{\left\|g\left(a_{y}^{k}\right)-R^{2^{k}}D-u^{k+1}+\frac{\eta^{k}}{\beta }\right\|}^{2}\\ +\langle \beta \nabla g\left(a_{y}^{k}\right)\left(g\left(a_{y}^{k}\right)-R^{2^{k}}D-u^{k+1}+\frac{\eta^{k}}{\beta }\right),a_{y}-a_{y}^{k}\rangle \end{array}$$

With the use of Formula (42), we obtain

(43) $$a_{y}^{k+1}=a_{y}^{k}-\frac{\beta \nabla g\left(a_{y}^{k}\right)}{\mu }\left(g\left(a_{y}^{k}\right)-R^{2^{k}}D-u^{k+1}+\frac{\eta^{k}}{\beta }\right)$$

where $\mu \geq \beta {\left\|\nabla g\left(a_{y}^{k}\right)\right\|}^{2}$.

**3.**  **Computing** $R^{2k+1}$

If $\varphi \left(x\right)=\frac{1}{2}{\left\|x\right\|}^{2}$, based on Formula (40), we obtain

(44) $$\Delta \varphi_{2}\left(R^{2},R^{2^{k}}\right)=\frac{1}{2}\left\|R^{2}-R^{2^{k}}\right\|$$

Formula (37) is equivalent to

(45) $$R^{2^{k+1}}=\underset{R^{2}\in {\mathbb{R}}^{m}}{\arg\min}R^{2}+\frac{\beta }{2}{\left\|g\left(a_{y}^{k+1}\right)-R^{2}D-u^{k+1}+\frac{\eta^{k}}{\beta }\right\|}^{2}+\frac{1}{2}{\left\|R^{2}-R^{2^{k}}\right\|}^{2}$$

Formula (45) represents a convex quadratic programming problem. By solving the following Equation (46), we also solve Formula (45):

(46) $$0=1-\beta D^{T}\left(g\left(a_{y}^{k+1}\right)-R^{2}D-u^{k+1}+\frac{\eta^{k}}{\beta }\right)+R^{2}-R^{2^{k}}$$

The value of $R^{2^{k+1}}$ is directly updated using the following formula:

(47) $$R^{2^{k+1}}=\frac{\left(\beta D^{T}\left(g\left(a_{y}^{k+1}\right)-u^{k+1}+\frac{\eta^{k}}{\beta }\right)-1+R^{2^{k}}\right)}{\left(1+\beta D^{T}D\right)}$$

**4.**  **Computing** $\eta^{k+1}$

$\eta^{k+1}$ can be calculated using Formula (38). When $\eta^{k+1}=0$, the Lagrange multiplier is removed.

Base on above analysis, the framework of our method can be summarized in Algorithm 2.
**Algorithm 2:** Fast ADMM Algorithm$\mathrm{Input}:\;K_{a},\;K_{b},\;D$$\mathrm{Output}:\;\left(R^{2^{k}},a_{y}^{k}\right)$$1:\;\mathrm{Take}\;\mathrm{an}\;\mathrm{initial}\;\mathrm{point}\;\left(R^{2^{0}},a_{y}^{0},u^{0},\eta^{0}\right)$, and set β$\;\mathrm{and}\;k_{MAX}$.$2:\;\mathrm{While}\;\mathrm{the}\;\mathrm{stop}\;\mathrm{condition}\;\mathrm{is}\;\mathrm{not}\;\mathrm{satisfied}\;\mathrm{and}\;k\leq k_{MAX}$ perform the following $3:\;\;\;\mathrm{Calculate}\;a_{y}^{k}$ according to Formula (43).$4:\;\;\;\mathrm{Calculate}\;R^{2^{k}}$ according to Formula (47). 5:   If the proximal operator of ϕ(u) has an explicit expression, use Formula (20) for the$\;\mathrm{calculation}\;u^{k}$6:   If the proximal operator of ϕ(u) does not have an explicit expression, use$\mathrm{Algorithm}\;1\;\mathrm{for}\;\mathrm{the}\;\mathrm{calculation}\;u^{k}$$7:\;\;\;\mathrm{Calculate}\;\eta^{k}$ according to Formula (38). 8:   Update k=k+1.9: End10: $\mathrm{Output}\;\;\mathrm{the}\;\mathrm{final}\;\mathrm{solution}\;\left(R^{2^{k}},a_{y}^{k}\right)$.

### 5.2. Global Convergence Analysis of the Fast ADMM Algorithm

In this section, we provide a convergence analysis of the fast ADMM algorithm. Specifically, the convergence of the algorithm is discussed using Lemma 7, and Lemma 7 is proven. According to Formulas (35)–(38), it can be observed that the optimality conditions for each update iteration of the fast ADMM algorithm can be written as follows:

(48) $$\{\begin{array}{c} 0\in \partial CL_{\phi }\left(u^{k+1}\right)-\eta^{k}-\beta \left(g\left({a_{y}}^{k}\right)-R^{2^{k}}D-u^{k+1}\right)\\ 0=\mu \left(a_{y}^{k+1}-a_{y}^{k}\right)-\beta \nabla g\left(a_{y}^{k}\right)\left(g\left(a_{y}^{k}\right)-R^{2^{k}}D-u^{k+1}+\frac{\eta^{k}}{\beta }\right)\\ 0=1-D^{T}\eta^{k}-\beta D^{T}\left(g\left({a_{y}}^{k+1}\right)-R^{2^{k+1}}D-u^{k+1}\right)+R^{2^{k+1}}-R^{2^{k}}\\ \eta^{k+1}=\eta^{k}+\beta \left(g\left({a_{y}}^{k+1}\right)-R^{2^{k+1}}D-u^{k+1}\right) \end{array}$$

where $\nabla g\left(a_{y}\right)=2D{a_{y}}^{T}K-2{K_{b}}^{T}$.

**Lemma 4.**  *Assume* $w^{k}=\left(R^{2^{k}},a_{y}^{k},u^{k},\eta^{k}\right)$ *is a sequence generated using the fast ADMM algorithm; then, for any* k≥1 *, we obtain the following: *

(49) $$\begin{array}{r} L_{\beta }\left(R^{2^{k+1}},a_{y}^{k+1},u^{k+1},\eta^{k}\right)\leq L_{\beta }\left(R^{2^{k}},a_{y}^{k+1},u^{k+1},\eta^{k}\right)-\frac{\beta }{2}{\left\|R^{2^{k+1}}D-R^{2^{k}}D\right\|}^{2}\\ -\frac{1}{2}{\left\|R^{2^{k+1}}-R^{2^{k}}\right\|}^{2}-\frac{1}{2}{\left\|R^{2^{k}}-R^{2^{k-1}}\right\|}^{2}\;\; \end{array}$$

*where* $\lambda_{\min}\left(DD^{T}\right)$ *represents the strictly positive minimum eigenvalue of* $DD^{T}$ *.*

**Proof of Lemma 4.**  According to the optimality conditions of the iteration, the following equation is relevant:

$$D^{T}\eta^{k+1}=1+R^{2^{k+1}}-R^{2^{k}}$$

According to the Cauchy–Schwarz inequality, it follows that

$$\begin{array}{l} \lambda_{\min}\left(DD^{T}\right){\left\|\eta^{k+1}-\eta^{k}\right\|}^{2}\leq {\left\|D^{T}\eta^{k+1}-D^{T}\eta^{k}\right\|}^{2}={\left\|R^{2^{k+1}}-R^{2^{k}}-\left(R^{2^{k}}-R^{2^{k-1}}\right)\right\|}^{2}\\ \;\;\leq {\left\|R^{2^{k+1}}-R^{2^{k}}\right\|}^{2}+{\left\|R^{2^{k}}-R^{2^{k-1}}\right\|}^{2} \end{array}$$

Thus,

$$\frac{1}{\beta }{\left\|\eta^{k+1}-\eta^{k}\right\|}^{2}\leq \frac{1}{\lambda_{\min}\left(DD^{T}\right)\beta }{\left\|R^{2^{k+1}}-R^{2^{k}}\right\|}^{2}+\frac{1}{\lambda_{\min}\left(DD^{T}\right)\beta }{\left\|R^{2^{k}}-R^{2^{k-1}}\right\|}^{2}.$$

□

**Lemma 5.**  *Assume* $w^{k}=\left(R^{2^{k}},a_{y}^{k},u^{k},\eta^{k}\right)$ *is a sequence generated using the fast ADMM algorithm; then, for any* k≥1 *, the following is true: *

(50) $$\begin{array}{r} L_{\beta }\left(R^{2^{k+1}},a_{y}^{k+1},u^{k+1},\eta^{k}\right)\leq L_{\beta }\left(R^{2^{k}},a_{y}^{k+1},u^{k+1},\eta^{k}\right)-\frac{\beta }{2}{\left\|R^{2^{k+1}}D-R^{2^{k}}D\right\|}^{2}\\ -\frac{1}{2}{\left\|R^{2^{k+1}}-R^{2^{k}}\right\|}^{2}-\frac{1}{2}{\left\|R^{2^{k}}-R^{2^{k-1}}\right\|}^{2}\;\; \end{array}$$

**Proof of Lemma 5.**  From the definition of the augmented Lagrangian function presented in Formula (34), it can be argued that

(51) $$\begin{array}{l} L_{\beta }\left(R^{2^{k+1}},a_{y}^{k+1},u^{k+1},\eta^{k}\right)-L_{\beta }\left(R^{2^{k}},a_{y}^{k+1},u^{k+1},\eta^{k}\right)\\ =R^{2^{k+1}}-R^{2^{k}}+\langle \eta^{k},\left(R^{2^{k}}-R^{2^{k+1}}\right)D\rangle +\frac{\beta }{2}{\left\|g\left(a_{y}^{k+1}\right)-R^{2^{k+1}}D-u^{k+1}\right\|}^{2}-\frac{\beta }{2}{\left\|g\left(a_{y}^{k+1}\right)-R^{2^{k}}D-u^{k+1}\right\|}^{2}\\ +\frac{1}{2}{\left\|R^{2^{k+1}}-R^{2^{k}}\right\|}^{2}-\frac{1}{2}{\left\|R^{2^{k}}-R^{2^{k-1}}\right\|}^{2} \end{array}$$

Formula (48) shows the following:

(52) $$\;g\left(a_{y}^{k+1}\right)-R^{2^{k}}D-u^{k+1}=\frac{1}{\beta }\left(\eta^{k+1}-\eta^{k}\right)+\left(R^{2^{k+1}}D-R^{2^{k}}D\right)$$

Therefore,

(53) $$\begin{array}{l} \frac{\beta }{2}{\left\|\varphi \left(a_{y}^{k+1}\right)-R^{2^{k}}D-u^{k+1}\right\|}^{2}=\frac{\beta }{2}{\left\|\frac{1}{\beta }\left(\eta^{k+1}-\eta^{k}\right)+\left(R^{2^{k+1}}D-R^{2^{k}}D\right)\right\|}^{2}\\ =\frac{1}{2\beta }{\left\|\eta^{k+1}-\eta^{k}\right\|}^{2}+\frac{\beta }{2}{\left\|R^{2^{k+1}}D-R^{2^{k}}D\right\|}^{2}+\langle \eta^{k+1}-\eta^{k},R^{2^{k+1}}D-R^{2^{k}}D\rangle \end{array}$$

By substituting Formula (53) into (51), the following is achieved:

$$\begin{array}{l} L_{\beta }\left(R^{2^{k+1}},a_{y}^{k+1},u^{k+1},\eta^{k}\right)-L_{\beta }\left(R^{2^{k}},a_{y}^{k+1},u^{k+1},\eta^{k}\right)\\ =R^{2^{k+1}}-R^{2^{k}}+\langle \eta^{k},\left(R^{2^{k}}-R^{2^{k+1}}\right)D\rangle -\frac{\beta }{2}{\left\|R^{2^{k+1}}D-R^{2^{k}}D\right\|}^{2}-\langle \eta^{k+1}-\eta^{k},R^{2^{k+1}}D-R^{2^{k}}D\rangle \\ +\frac{1}{2}{\left\|R^{2^{k+1}}-R^{2^{k}}\right\|}^{2}-\frac{1}{2}{\left\|R^{2^{k}}-R^{2^{k-1}}\right\|}^{2}\\ =-\frac{\beta }{2}{\left\|R^{2^{k+1}}D-R^{2^{k}}D\right\|}^{2}+\langle 1-D^{T}\eta^{k+1},R^{2^{k+1}}-R^{2^{k}}\rangle +\frac{1}{2}{\left\|R^{2^{k+1}}-R^{2^{k}}\right\|}^{2}-\frac{1}{2}{\left\|R^{2^{k}}-R^{2^{k-1}}\right\|}^{2}\\ =-\frac{\beta }{2}{\left\|R^{2^{k+1}}D-R^{2^{k}}D\right\|}^{2}-\frac{1}{2}{\left\|R^{2^{k+1}}-R^{2^{k}}\right\|}^{2}-\frac{1}{2}{\left\|R^{2^{k}}-R^{2^{k-1}}\right\|}^{2} \end{array}$$

Thus, the proof is completed. □

**Lemma 6.**  *Assume* $w^{k}=\left(R^{2^{k}},a_{y}^{k},u^{k},\eta^{k}\right)$ *is a sequence generated using the fast ADMM algorithm; then, for* k≥1 *, we obtain the following: *

(54) $$\begin{array}{c} L_{\beta }\left(R^{2^{k}},a_{y}^{k+1},u^{k+1},\eta^{k}\right)\leq L_{\beta }\left(R^{2^{k}},a_{y}^{k},u^{k+1},\eta^{k}\right)+\frac{\beta {\left\|\nabla g\left(a_{y}^{k}\right)\right\|}^{2}}{2}{\left\|a_{y}^{k}-a_{y}^{k-1}\right\|}^{2}\\ -\frac{\mu }{2}{\left\|a_{y}^{k+1}-a_{y}^{k}\right\|}^{2}-\frac{\mu }{2}{\left\|a_{y}^{k}-a_{y}^{k-1}\right\|}^{2} \end{array}$$

**Proof of Lemma 6.**  The definition of the augmented Lagrangian function in Formula (34) shows the following:

(55) $$\begin{array}{l} L_{\beta }\left(R^{2^{k}},a_{y}^{k+1},u^{k+1},\eta^{k}\right)-L_{\beta }\left(R^{2^{k}},a_{y}^{k},u^{k+1},\eta^{k}\right)\\ =\frac{\beta }{2}{\left\|g\left(a_{y}^{k}\right)-R^{2^{k}}D-u^{k+1}+\frac{\eta^{k}}{\beta }\right\|}^{2}-\frac{\mu }{2}{\left\|a_{y}^{k+1}-a_{y}^{k}\right\|}^{2}-\frac{\mu }{2}{\left\|a_{y}^{k}-a_{y}^{k-1}\right\|}^{2}-\frac{\beta }{2}{\left\|g\left(a_{y}^{k-1}\right)-R^{2^{k}}D-u^{k+1}+\frac{\eta^{k}}{\beta }\right\|}^{2}\\ -\langle \beta \nabla g\left(a_{y}^{k-1}\right)\left(g\left(a_{y}^{k-1}\right)-R^{2^{k}}D-u^{k+1}+\frac{\eta^{k}}{\beta }\right),a_{y}^{k}-a_{y}^{k-1}\rangle \\ =\frac{\beta }{2}{\left\|g\left(a_{y}^{k}\right)-g\left(a_{y}^{k-1}\right)\right\|}^{2}-\frac{\mu }{2}{\left\|a_{y}^{k+1}-a_{y}^{k}\right\|}^{2}-\frac{\mu }{2}{\left\|a_{y}^{k}-a_{y}^{k-1}\right\|}^{2}+\beta \langle g\left(a_{y}^{k-1}\right)-R^{2^{k}}D-u^{k+1}+\frac{\eta^{k}}{\beta },g\left(a_{y}^{k}\right)-g\left(a_{y}^{k-1}\right)\rangle \\ -\langle \beta \nabla g\left(a_{y}^{k-1}\right)\left(g\left(a_{y}^{k-1}\right)-R^{2^{k}}D-u^{k+1}+\frac{\eta^{k}}{\beta }\right),a_{y}^{k}-a_{y}^{k-1}\rangle \end{array}$$

According to the first-order condition for convex functions, and since $g\left(a_{y}\right)$ is a convex function, we obtain the following:

(56) $$g\left(a_{y}^{k+1}\right)-g\left(a_{y}^{k}\right)\leq \langle \partial g\left(a_{y}^{k+1}\right),a_{y}^{k+1}-a_{y}^{k}\rangle$$

The substitution of Formula (56) yields the following:

$$\begin{array}{l} L_{\beta }\left(R^{2^{k}},a_{y}^{k+1},u^{k+1},\eta^{k}\right)-L_{\beta }\left(R^{2^{k}},a_{y}^{k},u^{k+1},\eta^{k}\right)\\ =\frac{\beta }{2}{\left\|g\left(a_{y}^{k}\right)-g\left(a_{y}^{k-1}\right)\right\|}^{2}-\frac{\mu }{2}{\left\|a_{y}^{k+1}-a_{y}^{k}\right\|}^{2}-\frac{\mu }{2}{\left\|a_{y}^{k}-a_{y}^{k-1}\right\|}^{2}+\beta \langle g\left(a_{y}^{k-1}\right)-R^{2^{k}}D-u^{k+1}+\frac{\eta^{k}}{\beta },g\left(a_{y}^{k}\right)-g\left(a_{y}^{k-1}\right)\rangle \\ -\langle \beta \nabla g\left(a_{y}^{k-1}\right)\left(g\left(a_{y}^{k-1}\right)-R^{2^{k}}D-u^{k+1}+\frac{\eta^{k}}{\beta }\right),a_{y}^{k}-a_{y}^{k-1}\rangle \\ \leq \frac{\beta }{2}{\left\|g\left(a_{y}^{k}\right)-g\left(a_{y}^{k-1}\right)\right\|}^{2}-\frac{\mu }{2}{\left\|a_{y}^{k+1}-a_{y}^{k}\right\|}^{2}-\frac{\mu }{2}{\left\|a_{y}^{k}-a_{y}^{k-1}\right\|}^{2}\\ \leq \frac{\beta {\left\|\nabla g\left(a_{y}^{k}\right)\right\|}^{2}}{2}{\left\|a_{y}^{k}-a_{y}^{k-1}\right\|}^{2}-\frac{\mu }{2}{\left\|a_{y}^{k+1}-a_{y}^{k}\right\|}^{2}-\frac{\mu }{2}{\left\|a_{y}^{k}-a_{y}^{k-1}\right\|}^{2} \end{array}$$

Thus, the proof is complete. □

**Lemma 7.**  *Assume* $w^{k}=\left(R^{2^{k}},a_{y}^{k},u^{k},\eta^{k}\right)$ *is a sequence generated using the fast ADMM algorithm; if* $\mu \geq \beta {\left\|\nabla g\left(a_{y}^{k}\right)\right\|}^{2}$ *and* $\frac{1}{\lambda_{\min}\left(DD^{T}\right)\beta }\leq \frac{1}{2}$ *, then for any* k≥1 *, we obtain the following: *

(57) $$L_{\beta }\left(R^{2^{k+1}},a_{y}^{k+1},u^{k+1},\eta^{k+1}\right)\leq L_{\beta }\left(R^{2^{k}},a_{y}^{k},u^{k},\eta^{k}\right)-\frac{\mu }{2}{\left\|a_{y}^{k+1}-a_{y}^{k}\right\|}^{2}-\frac{\beta }{2}{\left\|R^{2^{k+1}}D-R^{2^{k}}D\right\|}^{2}$$

**Proof of Lemma 7.**  The definition of the augmented Lagrangian function in Formula (36) shows the following:

$$\begin{array}{c} L_{\beta }\left(R^{2^{k+1}},a_{y}^{k+1},u^{k+1},\eta^{k+1}\right)=L_{\beta }\left(R^{2^{k+1}},a_{y}^{k+1},u^{k+1},\eta^{k}\right)+\langle \eta^{k+1}-\eta^{k},\varphi \left(a_{y}^{k+1}\right)-R^{2^{k+1}}D-u^{k+1}\rangle \\ =L_{\beta }\left(R^{2^{k+1}},a_{y}^{k+1},u^{k+1},\eta^{k}\right)+\frac{1}{\beta }{\left\|\eta^{k+1}-\eta^{k}\right\|}^{2} \end{array}$$

Since $L_{\beta }\left(R^{2^{k}},a_{y}^{k},u,\eta^{k}\right)$ is a global minimum with respect to the variable $u^{k+1}$, then

$$L_{\beta }\left(R^{2^{k}},a_{y}^{k},u^{k+1},\eta^{k}\right)\leq L_{\beta }\left(R^{2^{k}},a_{y}^{k},u^{k},\eta^{k}\right)$$

The combination of Lemmas 5 and 6 produces the following:

$$\begin{array}{l} L_{\beta }\left(R^{2^{k+1}},a_{y}^{k+1},u^{k+1},\eta^{k+1}\right)=L_{\beta }\left(R^{2^{k+1}},a_{y}^{k+1},u^{k+1},\eta^{k}\right)+\frac{1}{\beta }{\left\|\eta^{k+1}-\eta^{k}\right\|}^{2}\\ =L_{\beta }\left(R^{2^{k}},a_{y}^{k+1},u^{k+1},\eta^{k}\right)-\frac{\beta }{2}{\left\|R^{2^{k+1}}D-R^{2^{k}}D\right\|}^{2}+\frac{1}{\beta }{\left\|\eta^{k+1}-\eta^{k}\right\|}^{2}-\frac{1}{2}{\left\|R^{2^{k+1}}-R^{2^{k}}\right\|}^{2}-\frac{1}{2}{\left\|R^{2^{k}}-R^{2^{k-1}}\right\|}^{2}\\ \leq \;L_{\beta }\left(R^{2^{k}},a_{y}^{k},u^{k},\eta^{k}\right)-\frac{\beta }{2}{\left\|R^{2^{k+1}}D-R^{2^{k}}D\right\|}^{2}-\frac{1}{2}{\left\|R^{2^{k+1}}-R^{2^{k}}\right\|}^{2}-\frac{1}{2}{\left\|R^{2^{k}}-R^{2^{k-1}}\right\|}^{2}+\frac{1}{\beta }{\left\|\eta^{k+1}-\eta^{k}\right\|}^{2}\\ +\frac{\beta {\left\|\nabla g\left(a_{y}^{k}\right)\right\|}^{2}}{2}{\left\|a_{y}^{k}-a_{y}^{k-1}\right\|}^{2}-\frac{\mu }{2}{\left\|a_{y}^{k+1}-a_{y}^{k}\right\|}^{2}-\frac{\mu }{2}\left\|a_{y}^{k}-a_{y}^{k-1}\right\| \end{array}$$

When $\mu \geq \beta {\left\|\nabla g\left(a_{y}^{k}\right)\right\|}^{2}$ and $\frac{1}{\lambda_{\min}\left(DD^{T}\right)\beta }\leq \frac{1}{2}$, it follows that

$$L_{\beta }\left(R^{2^{k+1}},a_{y}^{k+1},u^{k+1},\eta^{k+1}\right)\leq L_{\beta }\left(R^{2^{k}},a_{y}^{k},u^{k},\eta^{k}\right)-\frac{\mu }{2}{\left\|a_{y}^{k+1}-a_{y}^{k}\right\|}^{2}-\frac{\beta }{2}{\left\|R^{2^{k+1}}D-R^{2^{k}}D\right\|}^{2}$$

When the conditions of $\mu \geq \beta {\left\|\nabla g\left(a_{y}^{k}\right)\right\|}^{2}$ and $\frac{1}{\lambda_{\min}\left(DD^{T}\right)\beta }\leq \frac{1}{2}$ are met, according to Lemma 7, $L_{\beta }\left(R^{2},a_{y},u,\eta \right)$ monotonically decreases, thus causing the fast ADMM algorithm to converge. □

### 5.3. Fast ADMM Algorithm Termination Conditions

Based on Formula (35), it can be observed that the $u^{k+1}$ that minimizes $L_{\beta }\left(R^{2^{k}},a_{y}^{k},u,\eta^{k}\right)$ can be derived as follows:

(58) $$\begin{array}{l} 0\in \partial CL_{\phi }\left(u^{k+1}\right)-\beta \left(g\left(a_{y}^{k}\right)-R^{2^{k}}D-u^{k+1}+\frac{\eta^{k}}{\beta }\right)\\ =\partial CL_{\phi }\left(u^{k+1}\right)-\eta^{k+1}-\beta \left(g\left(a_{y}^{k+1}\right)-g\left(a_{y}^{k}\right)\right)-\beta \left(R^{2^{k+1}}D-R^{2^{k}}D\right) \end{array}$$

where

(59) $$\beta \left(g\left(a_{y}^{k+1}\right)-g\left(a_{y}^{k}\right)\right)+\beta \left(R^{2^{k+1}}D-R^{2^{k}}D\right)\in \partial CL_{\phi }\left(u^{k+1}\right)-\eta^{k+1}$$

Thus, we obtain the following equation:

(60) $$S_{1}^{k+1}=\beta \left(g\left(a_{y}^{k+1}\right)-g\left(a_{y}^{k}\right)\right)+\beta \left(R^{2^{k+1}}D-R^{2^{k}}D\right)$$

Equation (60) represents the residual value of the dual feasibility condition. Hence, $S_{1}^{k+1}$ represents the dual residual of iteration.

(61) $$\;r_{1}^{k+1}=K_{a}-2K_{b}a_{y}^{k+1}+a_{y}^{k+1^{T}}Ka_{y}^{k+1}D-R^{2^{k+1}}D-u^{k+1}$$

(62) $$\;r_{2}^{k+1}=1-\eta^{k+1^{T}}D$$

(63) $$\;r_{3}^{k+1}=\left(2D{a_{y}^{k+1}}^{T}K-2{K_{b}}^{T}\right)\eta^{k+1^{T}}$$

Equations (61)–(63) represent the primal residuals of iteration. The KKT conditions of problem (32) consist of four components, corresponding to the primal and dual residuals. These two types of residuals gradually converge to zero with the use of the fast ADMM algorithm.

Both the primal and dual residuals must be sufficiently small, meeting the conditions presented below, for the termination of the use of the fast ADMM algorithm:

(64) $$\max\left\{\begin{array}{l} \frac{\left\|r_{1}^{k}\right\|}{\max\left\{\left\|2K_{b}a_{y}+a_{y}^{T}Ka_{y}D-R^{2}D\right\|,\left\|u\right\|,\left\|K_{a}\right\|\right\}},\frac{\left\|S_{1}^{k}\right\|}{\left\|\eta^{k^{T}}\right\|},\frac{\left\|r_{2}^{k}\right\|}{\max\left\{1,\left\|\eta^{k^{T}}D\right\|\right\}},\\ \frac{\left\|r_{3}^{k}\right\|}{\max\left\{\left\|2D{a_{y}^{k+1}}^{T}K-2{K_{b}}^{T}\right\|,\left\|2\eta^{k+1^{T}}\right\|\right\}} \end{array}\right\}\leq \varepsilon^{tol}$$

where $\varepsilon^{tol}>0$. Typically, $\varepsilon^{tol}$ is selected so that $10^{-4}\leq \varepsilon^{tol}\leq 10^{-3}$; in this study, $\varepsilon^{tol}=10^{-3}$. This ensures that the algorithm terminates when the solution’s accuracy is within an acceptable range.

## 6. Experiment

In this section, we describe extensive experiments conducted on various datasets to validate the effectiveness and robustness of the three truncated loss function SVDD algorithms proposed in this study, which are presented below. The experimental datasets consisted of synthetic and several UCI datasets. We also compared hinge loss SVDD [8], DW-SVDD [20], R-SVDD [19], and GL-SVDD [16] algorithms to validate their performance.

1. $\phi_{R}$-SVDD: SVDD algorithm with a truncated generalized ramp loss function;
2. $\phi_{f}$-SVDD: SVDD algorithm with a truncated binary cross entropy loss function;
3. $\phi_{L}$-SVDD: SVDD algorithm with a truncated linear exponential loss function.

### 6.1. Experimental Setup

This subsection introduces the evaluation metrics, kernels, and parameter settings used in the experiments.

#### 6.1.1. Evaluation Metrics

We used G-mean and $F_{1}$-score as evaluation metrics to assess the performance of the different methods described in this study [30,39,40,41]. They are based on TP, FN, FP, and TN, where TP denotes the number of target data predicted as target data, FN denotes the number of target data predicted as outlier data, FP denotes the number of outlier data predicted as target data, and TN denotes the number of outlier data predicted as outlier data.

(65) G−mean=Recall×Specificity

(66) F1−score=2×Precision×RecallPrecision+Recall

where Recall=TP/(TP+FN), Specificity=TN/(TN+FP), and Precision=TP/(TP+FP). It can be seen from (65) and (66) that G-mean can provide a good balance between recall and specificity, and $F_{1}$-score provides a good balance between precision and recall. Hence, both of them can measure the performance of the proposed method, comprehensively.

#### 6.1.2. Kernels

The Gaussian kernel function has the best mapping ability and is the most practical method, commonly used to handle the classification problems of nonlinear data, and we used it when conducting our experiments. The definition of the Gaussian kernel function is as follows:

(67) $$K\left(x_{i},x_{j}\right)=\exp\left(-\frac{{\left\|x_{i}-x_{j}\right\|}^{2}}{2\sigma^{2}}\right)$$

#### 6.1.3. Parameter Configuration

To ensure a fair comparison, the parameters for each method were selected using the grid search method. For all methods, the regularization parameter c was selected from {0.05,0.1,0.3,0.5,0.7,0.9,1,2,5,10}. Arin Chaudhuri [42] has demonstrated that there exists a σ in the interval $\left[\min\left\{\sqrt{{\left\|x_{i}-x_{j}\right\|}^{2}},i\neq j\right\}/\sqrt{3},\max\left\{\sqrt{{\left\|x_{i}-x_{j}\right\|}^{2}},i\neq j\right\}/\sqrt{3}\right]$ that maximizes the SVDD optimization objective function. Therefore, the range for the kernel parameter σ can be set as: $[\min\left\{\sqrt{{\left\|x_{i}-x_{j}\right\|}^{2}},i\neq j\right\}/\sqrt{3},$ $\max\left\{\sqrt{{\left\|x_{i}-x_{j}\right\|}^{2}},i\neq j\right\}/\sqrt{3}]$.

The DW-SVDD and GL-SVDD algorithms locate the k-nearest neighbors in the feature space, with the parameter k selected from {5,10,15,20}. All of the truncated loss function SVDD algorithms select the parameter λ from {0.1,0.2,0.5,1}, $\phi_{R}$-SVDD selects parameter v from {0.1,0.3,0.5,1,5}, and $\phi_{f}$-SVDD selects parameter θ from {0.1,0.3,0.5,1,5}. The parameter a for $\phi_{L}$-SVDD is selected from {1,5,10}.

### 6.2. Synthetic Datasets with Noise

In this study, we constructed two synthetic datasets: circular and banana-shaped, to test the robustness of the three proposed truncated loss function support vector data description (SVDD) algorithms. To verify the robustness of the algorithms in handling different types of noise, we introduced two types of noise:

- Neighboring noise: Noise points are randomly distributed near the normal samples but do not completely overlap with the normal samples, forming a more discrete distribution characteristic. This noise simulates the common boundary ambiguity in practical applications.
- Regional noise: Noise points are randomly distributed within a specified area, forming sparse clusters. This noise simulates possible local anomalies in practical applications.

**Figure 1:**
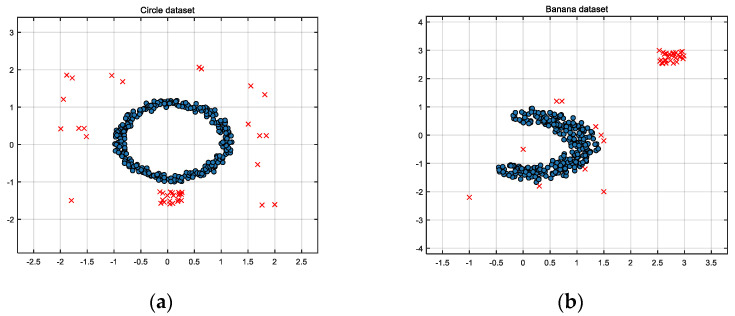
Two synthetic datasets, where blue samples are normal and the samples marked with a red cross represent noise. (**a**) Circular dataset. (**b**) Banana-shaped dataset.

The description of the synthetic dataset is given below:

1. Circular Dataset
  - Normal samples: This consists of 300 two-dimensional sample points distributed within a concentric circle, forming a normal distribution pattern.
  - Noise samples: This consists of 40 noise points, divided into two types: 20 noise points randomly distributed near the normal sample points, showing a discrete distribution characteristic, and another 20 noise points randomly distributed within a specified area, forming sparse clusters.
  - Illustration: Figure 1a shows the circular dataset, where blue points represent normal samples, and red points represent noise.
2. Banana-shaped Dataset
  - Normal samples: This consists of 300 two-dimensional sample points distributed along curved lines, resembling a banana shape.
  - Noise samples: This consists of 40 noise points, divided into two types: 10 noise points randomly distributed near the banana-shaped normal sample points, and another 30 noise points randomly distributed within a specified area, forming sparse clusters.
  - Illustration: Figure 1b shows the banana-shaped dataset, where blue points represent normal samples, and red points represent noise samples.

By choosing these noise distributions and quantities, we aimed to simulate different real-world noise scenarios and test the robustness of the proposed algorithms under varying noise conditions.

**Figure 2:**
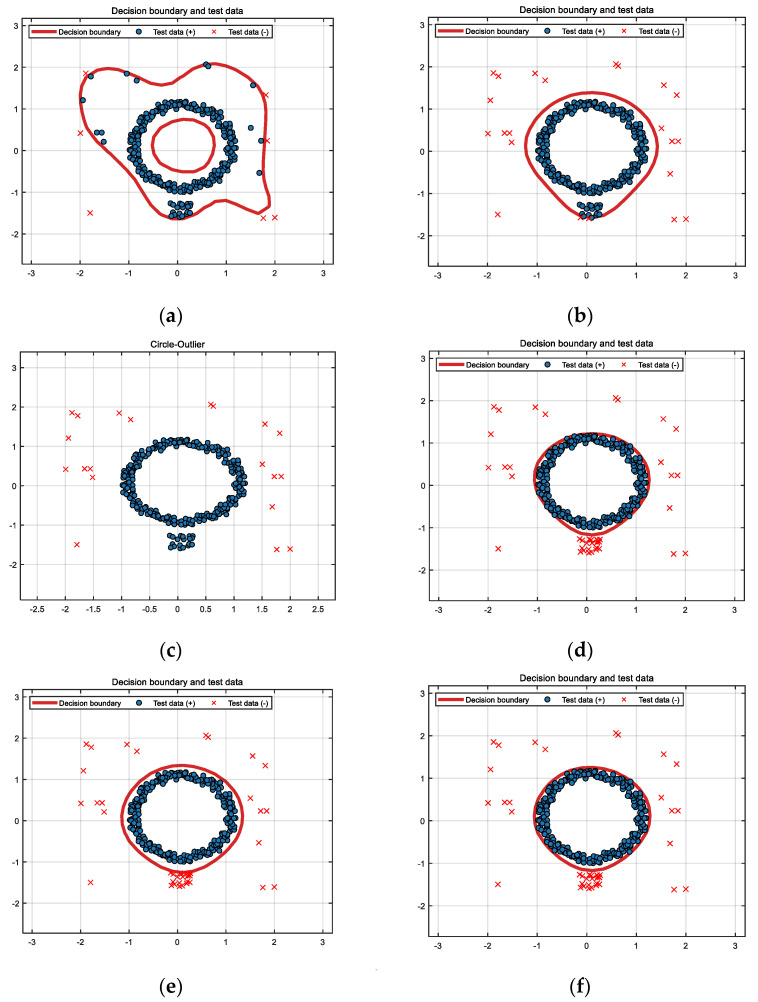
Classification results of six methods for circular dataset: (**a**) SVDD, (**b**) DW-SVDD, (**c**) DBSCAN, (**d**) $\phi_{R}$-SVDD, (**e**) $\phi_{f}$-SVDD, and (**f**) $\phi_{L}$-SVDD.

For circular outliers, the classification results of SVDD, DW-SVDD, DBSCAN, $\phi_{R}$-SVDD, $\phi_{f}$-SVDD, and $\phi_{L}$-SVDD algorithms for this dataset are presented in Figure 2. We can observe that the SVDD algorithm accurately identifies seven noise points as outliers, while misclassifying thirty-three noise points as normal values. Therefore, the performance of SVDD is severely affected by noise. Unlike SVDD, DW-SVDD accurately identified 22 noise points as outliers, while misclassifying 18 clustered noise points as normal values. DW-SVDD is based on a weighted idea where outliers are commonly sparse data; hence, a smaller weight is designated to these sparse data, which are then excluded from the sphere. The weighted method struggles to exclude clustered noise since 20 clustered noise points are present in the circular outliers. DBSCAN accurately identified the 20 sparsely distributed noise points but incorrectly classified the 20 clustered noise points as normal values. Therefore, DBSCAN cannot accurately identify clustered noise. The $\phi_{R}$-SVDD, $\phi_{f}$-SVDD, and $\phi_{L}$-SVDD algorithms successfully identified all of the noise points, and their classification boundaries encompassed all of the target samples.

**Figure 3:**
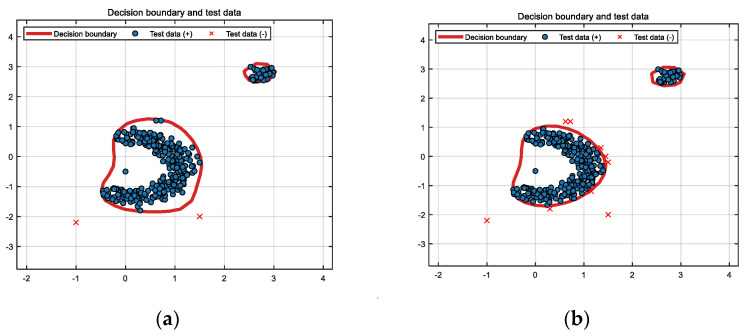

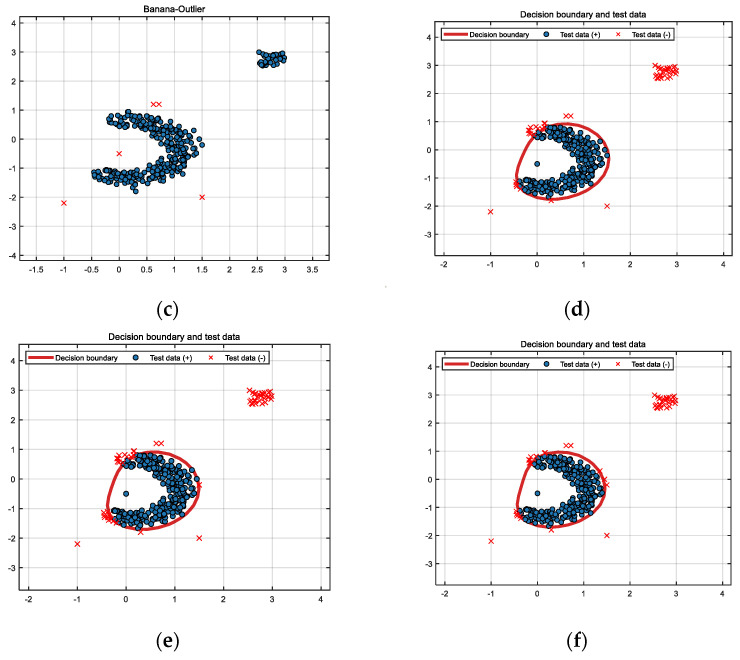
Classification results of six methods for banana-shaped dataset: (**a**) SVDD, (**b**) DW-SVDD, (**c**) DBSCAN, (**d**) $\phi_{R}$-SVDD, (**e**) $\phi_{f}$-SVDD, and (**f**) $\phi_{L}$-SVDD.

The classification results of the SVDD, DW-SVDD, DBSCAN, $\phi_{R}$-SVDD, $\phi_{f}$-SVDD, and $\phi_{L}$-SVDD algorithms for the banana-shaped dataset are presented in Figure 3. It can be observed that SVDD only identifies two noise points, while misclassifying the remaining noise points as normal values. DW-SVDD accurately differentiated nine noise points distributed around the banana data, while misclassifying the remaining noise points as normal values. $\phi_{R}$-SVDD accurately identified 35 noise points as outliers, while misclassifying the remaining noise points as normal values, and erroneously classifying 16 target values as outliers. $\phi_{f}$-SVDD accurately identified 36 noise points as outliers, while misclassifying the remaining noise points as normal values, and erroneously classifying 14 target values as outliers. $\phi_{L}$-SVDD accurately identified 38 noise points as outliers, while misclassifying the remaining noise points as normal values, and erroneously classifying 13 target values as outliers.

From the experiments on the two synthetic datasets, it can be seen that the DBSCAN algorithm is very effective in handling obvious noise, but it cannot accurately identify noise when the noise is less obvious or close to normal data. DW-SVDD and DBSCAN methods cannot accurately identify sparse clustered noise, whereas $\phi_{R}$-SVDD, $\phi_{f}$-SVDD, and $\phi_{L}$-SVDD can effectively identify clustered noise in the dataset. Figure 1 and Figure 2 show that the classification boundaries of $\phi_{R}$-SVDD, $\phi_{f}$-SVDD, and $\phi_{L}$-SVDD are tighter and smoother than SVDD and DW-SVDD methods. Therefore, it can be determined that the three truncated loss function SVDD algorithms proposed in this study are more robust to noise in both synthetic datasets.

### 6.3. UCI Datasets with the Presence of Noise

To further validate the effectiveness of the proposed method, we used eight standard datasets obtained from the UCI machine learning repository to conduct our experiments [43]. For each standard dataset, one class of samples was used as normal data, and the remaining classes were used as outlier data. Moreover, to eliminate the impact of data scale, each feature of every dataset was normalized to [0,1]. Grid parameter optimization was used to determine the optimal parameters in all of the experiments, and the average value of ten repetitions of each experiment was the final result. For each dataset, parts of the target and non-target data were randomly selected for the training set, and the remaining target and non-target data were selected for the test set.

#### 6.3.1. Training Dataset without Non-Target Data

For each dataset, 70% of the normal data were randomly selected for the training set, and noise-contaminated data were added to the training set. Noise data were created by changing the labels of non-target data from −1 to 1, with added noise data proportions of 10%, 20%, 30%, 40%, and 50% non-target data, and the test set consisted of the remaining target and non-target data.

Table 1 presents the G-mean averages for different methods across 10 trials, with the best results presented in bold. In 40 experimental datasets, $\phi_{R}$-SVDD and $\phi_{f}$-SVDD achieved higher G-means on 35 datasets compared to benchmark methods, while $\phi_{L}$-SVDD achieved higher G-means on 34 datasets. As the proportion of noise-contaminated data increases, i.e., more data are contaminated by noise, the classification accuracy of all models generally declines. It can be observed that, as the proportion of noise-contaminated data increases, the $\phi_{R}$-SVDD, $\phi_{f}$-SVDD, and $\phi_{L}$-SVDD algorithms show less of a decline in accuracy compared to the other algorithms. Taking the Iris dataset as an example, when the proportion of noise-contaminated data r increases from 0.1 to 0.5, the G-means of $\phi_{R}$-SVDD,$\phi_{f}$-SVDD, and $\phi_{L}$-SVDD decline by 13.02%, 13.02%, and 11.5%, while those of the DW-SVDD, R-SVDD, and GLE-SVDD decline by 19.55%, 16.6%, and 17.7%, respectively.

Table 2 shows the $F_{1}$-score averages of different methods over 10 trials. In the 40 experimental datasets, $\phi_{R}$-SVDD and $\phi_{f}$-SVDD achieved higher $F_{1}$-scores than the compared methods in 32 datasets, while $\phi_{L}$-SVDD had higher $F_{1}$-scores in 31 datasets. $\phi_{R}$-SVDD, $\phi_{f}$-SVDD, and $\phi_{L}$-SVDD demonstrate higher classification accuracy in most test datasets compared to current noise-resistant SVDD models (i.e., DW-SVDD, R-SVDD, and GL-SVDD), indicating that the use of a truncated loss function makes SVDD less sensitive to noise.

#### 6.3.2. Training Datasets with Non-Target Data

For each dataset, the training set was randomly selected to consist of 70% target and 20% non-target data, with added proportions of 10%, 20%, 30%, 40%, and 50% non-target data as noise data, and the test set included the remaining target and non-target data.

Table 3 presents the G-means from 10 trials for different methods, with the best results presented in bold. In 40 experimental datasets, $\phi_{R}$-SVDD and $\phi_{f}$-SVDD achieved higher G-means on 35 datasets compared to the benchmark methods, while $\phi_{L}$-SVDD achieved a higher G-mean on 33 datasets. As the data contaminated by noise increase, the $\phi_{R}$-SVDD, $\phi_{f}$-SVDD, and $\phi_{L}$-SVDD algorithms exhibit less of a decline in accuracy than the other algorithms. $\phi_{R}$-SVDD,$\phi_{f}$-SVDD, and $\phi_{L}$-SVDD present almost no decline in G-means for datasets such as Blood, Iris, and Haberman.

Table 4 shows the $F_{1}$-score averages of different methods over 10 trials. In the 40 experimental datasets, $\phi_{R}$-SVDD achieved higher $F_{1}$-scores than the compared methods in 35 datasets, $\phi_{f}$-SVDD performed better in 32 datasets, and $\phi_{L}$-SVDD in 31 datasets. From Table 1, Table 2, Table 3 and Table 4, it is evident that -SVDD, -SVDD, and -SVDD achieve better experimental performance than the other four methods, demonstrating superior noise resistance due to the use of the truncated loss function.

## 7. Conclusions

This study aimed to enhance the robustness and effectiveness of the SVDD algorithm. We propose a general framework for the truncated loss function of this algorithm, which uses bounded loss functions to mitigate the impact of outliers. Due to the non-differentiability of the truncated loss function, we employed the fast ADMM algorithm to solve the SVDD model with the truncated loss function, which handles truncated loss functions within a unified framework. In this context, the truncated generalized ramp, truncated binary cross entropy, and truncated linear exponential loss functions for the SVDD algorithm were constructed, and extensive experiments show that these three SVDD models exhibit more robustness than other SVDD models in most cases. However, this method still has the following shortcomings. Firstly, introducing the truncated loss function increases the complexity of model training, as some truncated loss functions cannot directly provide explicit expressions for neighboring point operators, requiring additional computational overhead. These factors may limit the application of the method to large-scale datasets. To overcome these limitations, future work can consider using a distributed computing framework to accelerate the training process of the ADMM algorithm. Secondly, the truncated loss function SVDD introduces new free parameters, which increases the time required for grid search parameter selection. When the data scale is large, the computation time for the grid search method may become unacceptable. To address this drawback, algorithms such as Bayesian optimization can be considered in the future to find the optimal parameters, further improving model performance and optimization efficiency. Finally, for extremely noisy data, the truncated loss function may not completely eliminate its impact, and the effect is limited. In this case, methods combining clustering algorithms such as DBSCAN can be adopted. First, clustering algorithms like DBSCAN can be used to preprocess the data and remove noise, and then the proposed method can be used to detect anomalies.

## Figures and Tables

**Table 1:** G-mean values for different methods for UCI datasets with noise (not containing non-target data).

Dataset	r	SVDD	DW-SVDD	R-SVDD	GL-SVDD	$\phi_{R}$-SVDD	$\phi_{f}$-SVDD	$\phi_{L}$-SVDD
Blood	10%	49.95	49.28	51.25	49.19	**52.79**	**52.79**	**54.82**
	20%	46.87	46.43	48.65	46.84	**50.21**	**50.21**	**51.5**
	30%	42.34	41.87	42.34	42.34	**45.96**	**45.96**	**52.41**
	40%	41.39	41.52	41.39	41.58	**45.06**	**45.06**	**51.50**
	50%	40.53	40.56	40.69	40.9	**42.6**	**42.6**	**50.72**
Balance scale	10%	73.70	73.8	73.91	73.64	**74.38**	**74.38**	**74.32**
	20%	**68.58**	**68.67**	**68.60**	**68.60**	66.62	66.62	67.19
	30%	62.66	**64.26**	62.66	62.71	62.70	62.70	63.75
	40%	57.38	58.44	57.38	57.44	**58.60**	**58.60**	**64.32**
	50%	53.47	54.72	53.43	53.47	**56.51**	**56.51**	**61.13**
*Ecoli*	10%	77.5	77.48	80.63	76.28	**86.08**	**86.08**	**81.95**
	20%	70.85	67.02	70.26	70.85	**73.08**	**73.08**	**73.53**
	30%	67	62.77	67.22	67	**70.61**	**70.61**	**70.29**
	40%	64.75	60.37	64.75	64.75	**67.57**	**67.57**	**68.33**
	50%	59.94	55.18	59.87	59.94	**64.26**	**64.26**	**64.42**
Haberman	10%	**54.37**	**54.57**	53.36	**55.49**	54.06	54.06	52.09
	20%	54.85	55.01	54.87	54.93	**55.34**	**55.34**	**54.30**
	30%	53.38	53.58	54.86	53.54	**57.13**	**57.13**	**56.02**
	40%	52.54	52.31	52.73	52.45	**53.89**	**53.89**	**53.93**
	50%	50.12	49.74	49.95	50.09	**51.92**	**51.92**	**52.03**
Iris	10%	76.8	79.5	76.92	78.18	**80.74**	**80.74**	**79.52**
	20%	69.29	68.94	69.39	69.52	**71.13**	**71.13**	**70.97**
	30%	65.77	65.52	65.67	65.85	**70.46**	**70.46**	**69.46**
	40%	61.67	61.69	61.41	61.96	**65.46**	**65.46**	**65.84**
	50%	60.21	59.95	60.32	60.48	**67.72**	**67.72**	**68.02**
Wine	10%	79.76	79.76	79.02	79.76	**80.18**	**80.18**	**80.74**
	20%	71.66	73.59	73.02	72.32	**75.03**	**75.03**	**76.23**
	30%	68.73	70.11	71.49	69.04	**73.12**	**73.12**	**72.44**
	40%	64.3	64.25	64.21	64.01	**68.35**	**68.35**	**68.96**
	50%	64.03	63.93	64.06	63.09	**68.00**	**68.00**	**68.59**
Ionosphere	10%	84.22	84.43	85.08	84.22	**86.89**	**86.89**	**86.81**
	20%	82.98	83.3	82.89	83.22	**84.12**	**84.12**	**83.38**
	30%	81.94	82.2	81.35	81.89	**82.42**	**82.42**	**82.18**
	40%	**83.55**	**83.37**	80.58	**83.33**	83.14	83.14	82.86
	50%	**82.28**	**82.44**	77.87	**82.28**	81.96	81.96	82.06
Sonar	10%	56.46	56.89	56.16	57.22	**58.62**	**58.62**	**59.58**
	20%	53.46	51.48	53.46	53.46	**53.50**	**53.50**	**53.73**
	30%	49.15	47.17	49.15	49.15	**55.29**	**55.29**	**55.28**
	40%	52.48	50.04	52.48	52.48	**57.57**	**57.57**	**57.68**
	50%	45.22	43.31	45.22	45.22	**50.10**	**50.10**	**50.38**

**Table 2:** *F*_1_-score values of different methods for UCI datasets with noise (not containing non-target data).

Dataset	r	SVDD	DW-SVDD	R-SVDD	GL-SVDD	$\phi_{R}$-SVDD	$\phi_{f}$-SVDD	$\phi_{L}$-SVDD
Blood	10%	72.17	**73.13**	**73.18**	**73.21**	73.05	73.05	72.93
	20%	**68.98**	68.09	**69.12**	**68.85**	68.72	68.72	68.19
	30%	65.93	66.54	67.33	65.93	**68.42**	**68.42**	**68.44**
	40%	64.42	64.95	64.26	64.28	**66.25**	**66.25**	**65.95**
	50%	63.38	62.37	63.27	62.64	**65.25**	**65.25**	**64.06**
Balance scale	10%	**58.14**	**58.28**	**58.13**	**58.07**	56.67	56.48	57.01
	20%	50.57	**51.47**	50.19	**51.49**	50.74	50.74	51.14
	30%	48.61	48.92	48.63	48.65	**49.75**	**49.75**	**49.58**
	40%	48.18	48.82	48.18	48.21	**53.02**	**53.02**	**53.65**
	50%	49.07	49.21	49.16	49.21	**49.81**	**49.81**	**53.48**
*Ecoli*	10%	60.55	60.48	60.51	55.27	**72.52**	**63.25**	**64.12**
	20%	47.43	50.81	46.49	50.81	**54.71**	**54.60**	**56.57**
	30%	49.30	47.04	43.51	49.30	**53.46**	**53.26**	**53.27**
	40%	50.95	49.43	46.08	50.95	**53.02**	**53.02**	**53.65**
	50%	50.49	50.58	49.20	50.58	**51.93**	**51.93**	**52.11**
Haberman	10%	57.98	58.28	56.01	**62.42**	57.63	58.15	57.79
	20%	**60.97**	60.17	59.67	60.29	60.65	60.27	59.76
	30%	62.18	61.95	62.36	62.23	**64.10**	**63.72**	**64.22**
	40%	63.87	64.41	62.04	64.02	**64.62**	**64.45**	**63.87**
	50%	56.66	58.02	57.51	58.25	**60.52**	**60.61**	**59.11**
Iris	10%	**66.07**	57.62	**68.75**	56.52	60.02	60.41	62.75
	20%	51.17	51.71	53.99	51.79	**55.66**	**55.66**	**55.79**
	30%	41.10	41.02	42.15	41.17	**49.74**	**49.74**	**51.22**
	40%	40.04	40.13	41.97	40.41	**44.94**	**44.94**	**45.79**
	50%	43.39	43.70	44.28	43.99	**49.97**	**49.97**	**50.34**
Wine	10%	60.87	63.87	62.77	53.66	**66.26**	**66.76**	**67.28**
	20%	44.97	43.48	41.97	43.94	**55.32**	**50.34**	**51.50**
	30%	42.46	43.91	43.33	42.81	**48.67**	**47.52**	**48.27**
	40%	42.06	42.45	42.56	41.94	**45.60**	**45.29**	**49.26**
	50%	46.64	46.61	46.24	45.98	**48.96**	**48.94**	**49.59**
Ionosphere	10%	80.87	81.09	81.29	80.87	**83.08**	**82.89**	**83.08**
	20%	80.40	80.03	80.74	80.31	**81.00**	**80.36**	**80.28**
	30%	79.46	79.76	79.98	79.40	**81.21**	**80.60**	**80.52**
	40%	81.52	81.93	81.26	81.88	**82.00**	**82.12**	**82.02**
	50%	81.85	81.67	81.48	81.67	**82.08**	**81.99**	**81.97**
Sonar	10%	**55.23**	54.3	**55.2**	54.6	54.17	54.17	55.14
	20%	51.43	52.25	52.25	52.35	**53.03**	**52.73**	52.13
	30%	48.73	47.78	48.73	48.73	**51.52**	**51.52**	**51.47**
	40%	51.16	50.34	51.16	51.16	**52.29**	**52.29**	**52.30**
	50%	48.26	49.08	49.08	49.08	**51.78**	**51.78**	**51.67**

**Table 3:** G-mean values of different methods on UCI datasets with noise (containing non-target data).

Dataset	r	SVDD	DW-SVDD	R-SVDD	GL-SVDD	$\phi_{R}$-SVDD	$\phi_{f}$-SVDD	$\phi_{L}$-SVDD
Blood	10%	53.18	51.66	52.05	53.66	52.29	52.29	**54.45**
	20%	50.82	50.91	51.65	51.57	**52.02**	**52.02**	**53.39**
	30%	49.72	49.19	51.13	48.78	**53.43**	**53.43**	**52.13**
	40%	51.00	49.23	50.16	48.43	**51.21**	**51.21**	**53.87**
	50%	48.04	48.72	47.89	46.88	**52.87**	**52.87**	**52.42**
Balance scale	10%	78.86	80.91	79.7	80.24	**81.25**	**81.25**	77.64
	20%	73.53	75.25	74.18	73.41	**76.07**	**76.07**	73.65
	30%	65.78	67.38	66.2	65.51	**68.09**	**68.09**	66.48
	40%	60.74	62.30	62.10	60.56	**67.11**	**67.11**	**65.78**
	50%	56.79	61.48	58.46	60.08	**68.24**	**68.24**	**64.86**
*Ecoli*	10%	82.1	82.36	83.95	83.08	**85.34**	**85.34**	**82.1**
	20%	77.46	77.48	80.02	77.08	**82.53**	**82.53**	**77.82**
	30%	69.55	67.74	68.52	70.88	**71.96**	**71.96**	**71.32**
	40%	74.09	70.07	71.19	72.08	**74.45**	**74.45**	**74.35**
	50%	66.93	63.38	64.87	63.58	**68.18**	**68.18**	**67.36**
Haberman	10%	55.59	55.41	54.81	55.51	**56.72**	**56.72**	**57.09**
	20%	58.66	58.88	54.12	58.80	**61.99**	**61.99**	**59.75**
	30%	55.13	55.22	54.2	55.47	**56.14**	**56.14**	**54.65**
	40%	51.83	52.00	57.63	50.38	**56.01**	**56.01**	**54.81**
	50%	55.64	55.70	55.26	55.52	**58.27**	**58.27**	**57.60**
Iris	10%	**80.7**	**81.45**	**81.22**	**80.38**	79.13	79.13	80.18
	20%	**79.01**	**78.31**	**78.75**	**77.94**	77.37	77.37	78.63
	30%	66.77	64.48	63.58	66.63	**71.12**	**71.12**	**70.78**
	40%	67.08	60.61	63.16	64.29	**70.91**	**70.91**	**70.47**
	50%	62.19	53.74	61.09	59.39	**67.59**	**67.59**	**67.63**
Wine	10%	81.54	84.11	83.77	81.54	**84.35**	**84.35**	**82.45**
	20%	79.12	81.44	80.24	79.12	**82.87**	**82.87**	**80.80**
	30%	71.03	69.15	70.02	71.03	**75.48**	**75.48**	**71.95**
	40%	69.28	67.97	67.75	69.28	**72.29**	**72.29**	**70.47**
	50%	64.77	66.80	66.46	64.77	**69.04**	**69.04**	**66.50**
Ionosphere	10%	86.45	86.79	88.66	87.26	**89.28**	**89.28**	**89.02**
	20%	84.84	86.01	86.38	86.38	**88.49**	**88.49**	**88.28**
	30%	84.25	85.0	85.09	85.18	**87.93**	**87.93**	**87.87**
	40%	82.58	83.13	83.37	83.37	**86.60**	**86.60**	**86.20**
	50%	77.26	78.56	78.54	78.62	**84.29**	**84.29**	**83.95**
Sonar	10%	57.24	58.34	**58.97**	**58.97**	58.66	58.66	58.70
	20%	59.23	59.25	**60.35**	**60.35**	60.21	60.21	59.79
	30%	54.51	54.76	56.03	56.03	**56.50**	**56.50**	**56.33**
	40%	55.86	55.86	58.93	58.93	**58.56**	**58.56**	**60.09**
	50%	52.21	53.14	53.92	53.92	**55.45**	**55.45**	**54.71**

**Table 4:** *F*_1_-score values of different methods for UCI datasets with noise (containing non-target data).

Dataset	r	SVDD	DW-SVDD	R-SVDD	GL-SVDD	$\phi_{R}$-SVDD	$\phi_{f}$-SVDD	$\phi_{L}-\mathrm{SVDD}$
Blood	10%	76.30	76.05	76.38	**77.20**	77.14	75.81	69.71
	20%	65.90	65.46	66.12	66.22	**73.71**	**70.79**	65.62
	30%	65.56	67.43	65.40	69.15	**69.32**	66.81	**68.44**
	40%	63.94	65.87	60.48	65.82	**70.09**	**66.07**	**66.46**
	50%	65.98	65.10	63.16	68.44	**80.57**	**68.88**	**69.03**
Balance scale	10%	65.77	68.66	66.89	67.52	**69.18**	66.75	66.74
	20%	63.48	62.23	62.34	61.64	**64.09**	61.90	**63.58**
	30%	58.62	58.54	56.99	61.23	**61.62**	**61.62**	59.06
	40%	64.74	63.67	63.67	63.16	**66.57**	**66.14**	**66.05**
	50%	68.25	68.78	66.92	68.02	**71.23**	**71.23**	**69.84**
*Ecoli*	10%	67.58	68.46	68.29	68.51	**76.37**	**68.84**	**68.58**
	20%	62.76	63.36	63.03	62.17	**70.01**	**69.01**	**68.16**
	30%	58.62	58.54	56.99	60.25	**61.39**	**61.75**	**61.11**
	40%	65.01	65.49	63.81	66.17	**67.89**	**67.86**	**67.67**
	50%	65.11	65.83	65.92	66.02	**66.86**	**66.17**	**67.92**
Haberman	10%	**61.22**	**61.07**	59.24	**61.12**	58.04	60.43	58.08
	20%	**66.36**	**66.28**	58.27	**66.19**	63.82	63.82	59.85
	30%	61.84	59.14	59.53	61.53	**64.97**	**65.48**	**64.48**
	40%	57.60	58.29	57.46	57.45	**63.08**	**64.17**	**62.35**
	50%	62.90	62.45	55.28	63.08	**66.34**	**70.63**	**63.96**
Iris	10%	61.66	62.84	63.26	61.13	61.91	**63.78**	60.98
	20%	55.14	55.39	56.23	53.85	**58.24**	**58.24**	**59.73**
	30%	52.68	50.74	53.21	52.68	**58.91**	**53.91**	**53.75**
	40%	54.74	51.56	53.88	55.98	**57.64**	**57.64**	**57.42**
	50%	54.19	57.43	58.42	58.47	**60.52**	**58.79**	**58.64**
Wine	10%	65.03	70.62	70.33	65.03	**70.90**	**68.82**	67.48
	20%	64.02	59.12	64.69	59.12	**62.37**	**59.29**	**59.35**
	30%	52.68	50.74	52.21	52.68	**58.91**	**53.91**	**53.75**
	40%	55.56	53.86	53.82	55.56	**58.78**	**56.90**	**56.17**
	50%	58.67	57.47	57.24	57.47	**60.52**	**59.79**	**58.94**
Ionosphere	10%	85.21	85.55	87.22	86.02	**87.95**	**87.95**	**87.64**
	20%	85.36	84.20	85.40	85.58	**87.48**	**87.48**	**87.22**
	30%	86.43	86.80	86.79	86.91	**88.45**	**88.45**	**88.38**
	40%	87.39	87.46	87.19	87.58	**88.95**	**88.8**	**88.67**
	50%	87.94	87.63	87.90	87.93	**89.24**	**89.07**	**88.92**
Sonar	10%	58.54	58.75	58.71	58.71	**59.05**	**58.88**	**59.12**
	20%	57.46	58.21	58.05	58.05	**58.74**	**58.74**	**58.18**
	30%	55.12	52.25	52.66	52.66	**60.26**	**59.52**	**56.25**
	40%	62.15	58.30	60.23	60.23	**63.52**	**63.52**	**62.38**
	50%	61.35	**64.91**	60.10	60.10	62.80	62.80	61.94

## Data Availability

No new data were created or analyzed in this study. Data sharing is not applicable to this article.
